# Machine-Learning-Driven Molecular Design and Structure–Property–Performance Relationships in Pharmaceutical Chemistry

**DOI:** 10.3390/molecules31122162

**Published:** 2026-06-19

**Authors:** Aisulu Zh. Kabdraisova, Almagul K. Umbetova, Gulfairuz Zh. Kairalapova, Yuliya A. Litvinenko, Larissa R. Sassykova, Nazym S. Yelibayeva, Gauhar Sh. Burasheva, Aliya E. Berganayeva, Zhanibek S. Assylkhanov, Meruyert D. Dauletova, Dmitriy Yu. Korulkin, Marzhan A. Baiburkutova, Aigerim M. Sadvakas

**Affiliations:** 1 Scientific Research Institute for New Chemical Technologies and Materials, Farabi University, 96a Tole Bi Str., 050012 Almaty, Kazakhstan; zhaksais@gmail.com; 2 Branch Office of the Republican State Enterprise “Infracos”, Abay Ave. 191, 050046 Almaty, Kazakhstan; 3 Department of Chemistry and Technology of Organic Substances, Chemistry of Natural Compounds and Polymers, Faculty of Chemistry and Chemical Technology, Farabi University, Al-Farabi Ave. 71, 050040 Almaty, Kazakhstan; nazym_yelibaeva@mail.ru (N.S.Y.); gauharbur@mail.ru (G.S.B.); aberganayeva@bk.ru (A.E.B.); zhanik1903@list.ru (Z.S.A.); dmd_09@inbox.ru (M.D.D.); dkorulkin@gmail.com (D.Y.K.); 4 Department of Physical Chemistry, Catalysis and Petrochemistry, Faculty of Chemistry and Chemical Technology, Farabi University, Al-Farabi Ave. 71, 050040 Almaty, Kazakhstan; larissa.rav@mail.ru; 5 JSC Scientific Center for Anti-Infectious Drugs, Al-Farabi Ave. 75B, 050060 Almaty, Kazakhstan; b_marzhan81@mail.ru (M.A.B.); a_sadvakas@mail.ru (A.M.S.)

**Keywords:** machine learning, pharmaceutical chemistry, molecular design, structure–property–performance relationships, retrosynthetic analysis, explainable artificial intelligence

## Abstract

This review examines the emerging role of machine learning (ML) in pharmaceutical chemistry, with emphasis on molecular design, synthetic feasibility, and structure–property–performance (SPP) relationships. By enabling pre-synthesis prediction of physicochemical properties, reaction pathways, and pharmaceutical performance, ML can reduce empirical trial-and-error experimentation and support more efficient exploration of chemical space. A structured narrative review design with PRISMA-aligned systematic search elements was used to evaluate 101 studies, enabling transparent literature identification, eligibility screening, and thematic synthesis across heterogeneous ML applications in pharmaceutical chemistry. This review examines structure–property relationships (SPRs) and property–performance relationships (PPRs), with emphasis on key pharmaceutical endpoints such as solubility, permeability, stability, dissolution, and bioavailability. An integrated SPP framework is proposed to connect molecular structure, intermediate properties, and final performance outcomes while incorporating retrosynthetic analysis and experimental feedback and closed-loop optimization. Recent frontier developments are also discussed, including molecular foundation models, multimodal language–graph models, diffusion-based molecular generation, E(3)-equivariant models, and MolMIM-like latent-space optimization. This review also covers co-folding and joint ligand–protein modeling, Boltz-2-like affinity prediction, AlphaFold 3-related biomolecular interaction modeling, and absorption, distribution, metabolism, excretion, and toxicity (ADMET) prediction. Key limitations include dataset leakage, benchmark inconsistency, assay variability, conformational and protonation-state effects, reproducibility challenges, regulatory constraints, and the gap between computational prediction and prospective experimental validation. Future progress is expected to depend on hybrid physics–ML models, uncertainty-aware prospective validation, autonomous experimentation, explainable artificial intelligence, and sustainability-aware molecular design. Overall, ML is evolving from a predictive tool into a chemically informed decision-support framework for rational, synthesis-aware, and experimentally validated pharmaceutical development.

## 1. Introduction

### 1.1. From Empirical Chemistry to Data-Driven Molecular Design

Pharmaceutical chemistry is shifting from empirical, trial-and-error molecular design toward data-driven, machine-learning-augmented strategies. Traditional workflows in medicinal and organic chemistry have relied on iterative structural modification followed by experimental validation. This empirical paradigm has enabled the discovery of many therapeutically relevant compounds, yet it remains constrained by limited exploration of chemical space and by uncertainties in synthetic feasibility. In practice, promising molecular designs are often deprioritized when their synthesis routes are complex, low-yielding, or poorly understood. This limitation highlights a persistent disconnect between molecular design and reaction-level feasibility.

Data-driven molecular design addresses these limitations by enabling predictive evaluation before synthesis. Quantitative structure–activity relationship approaches and related methodologies allow structure–function relationships to be inferred from large datasets, thereby reducing reliance on trial-and-error experimentation. More recently, these predictive frameworks have been extended to incorporate reaction knowledge, enabling simultaneous consideration of molecular desirability and synthetic accessibility. This shift is an important step toward rational, synthesis-aware pharmaceutical design [1,2,3]. At the same time, pharmaceutical chemistry continues to face major bottlenecks, including high attrition rates during drug development and the difficulty of navigating the vast pharmacologically relevant chemical space, which is estimated to exceed 10^60^ possible molecules [4,5]. These challenges limit the efficiency of traditional discovery pipelines and emphasize the need for approaches that prioritize promising candidates while accounting for molecular performance and synthetic feasibility. Accordingly, there is growing interest in ML frameworks that integrate de novo molecular design with SPP relationships and synthesis planning, thereby enabling the identification of chemically feasible and therapeutically relevant compounds [6,7,8].

### 1.2. Concept of Structure–Property–Performance (SPP) Relationships

The SPP framework provides a systematic basis for linking molecular design with pharmaceutical outcomes. In this paradigm, structure refers to molecular architecture, including functional groups, stereochemistry, and bonding patterns. These structural features determine properties, such as solubility, lipophilicity, stability, and reactivity. These properties, in turn, influence performance, which is defined by measurable outcomes such as dissolution behavior, formulation stability, and bioavailability in biological systems.

This hierarchical relationship aligns with established principles such as quantitative structure–property relationships but it extends beyond traditional property prediction by incorporating formulation and process-relevant endpoints. Importantly, SPP relationships must also account for synthetic feasibility. A molecule optimized for predicted properties may not translate into practical performance if it cannot be synthesized efficiently or reproducibly. Therefore, a comprehensive SPP framework requires integration with reaction design and synthesis planning, forming a structure–synthesis–property–performance continuum that connects molecular theory with experimental realization [4,9,10].

### 1.3. Role of Machine Learning in Modern Pharmaceutical Chemistry

ML-driven retrosynthesis and reaction prediction models can capture complex reactivity patterns across diverse chemical transformations, enabling rapid exploration of synthetic pathways that would be impractical to enumerate manually. These approaches are supported by large patent-derived reaction datasets, including the United States Patent and Trademark Office (USPTO) reaction corpus and USPTO-50K benchmark, as well as by template-free learning strategies that improve generalization across reaction classes [11,12,13].

Beyond property prediction, ML plays a central role in reaction design and synthesis planning. Data-driven retrosynthetic analysis allows for automated decomposition of target molecules into feasible precursors and reaction pathways. Models for reaction outcome prediction, yield estimation, and condition optimization further support the design of experimentally viable synthesis routes [11,14]. This integration of predictive modeling with synthetic planning is essential for translating computational designs into practical pharmaceutical candidates [6,14,15].

Despite these advances, limited chemical interpretability remains a major constraint. Many models operate as black boxes, providing predictions without mechanistic explanation. This limits their value for experimental chemists and creates challenges for validation and regulatory acceptance. Addressing this limitation requires interpretable ML approaches that are consistent with established principles of chemical reactivity and mechanism [14,16,17].

### 1.4. Scope and Contributions of This Review

**Figure 1:**
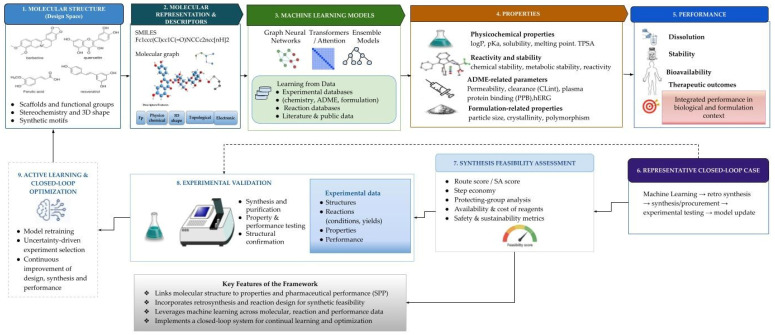
Integrated SPP–retrosynthesis framework for ML-driven pharmaceutical molecular design, linking molecular representation, property prediction, synthesis-feasibility assessment, experimental validation, and feedback-based model refinement. (Solid arrows indicate the primary molecular design and prediction workflow, whereas dashed arrows indicate feedback-based active-learning and model-refinement pathways).

This review focuses on the integration of ML with molecular design, synthetic feasibility, and SPP relationships in pharmaceutical chemistry, as schematically illustrated in Figure 1. The proposed framework provides a unified view of how molecular structures are converted into predictive representations, processed through ML models, and linked to both physicochemical properties and pharmaceutical performance. As shown in Figure 1, molecular structures are first encoded as descriptors, fingerprints, strings, graphs, or 3D representations, which serve as inputs for predicting key properties such as solubility, stability, and permeability. These predicted properties directly influence performance metrics, including dissolution behavior and bioavailability. In parallel, retrosynthetic analysis and reaction design modules evaluate synthetic feasibility by identifying viable disconnections, reaction pathways, and optimal conditions. This synthesis-aware layer helps ensure that designed molecules are not only theoretically promising but also experimentally realizable. Figure 1 also highlights synthesis feasibility assessment and experimental validation, where predicted molecules are synthesized or procured and then tested to generate high-quality data. These data can be reintegrated into the modeling pipeline through active learning, enabling continuous refinement of molecular design and predictive accuracy. To make this workflow operational, Figure 1 can be interpreted through representative ML-guided molecular design scenarios. For example, a candidate molecule may first be encoded using descriptors, SMILES/SELFIES strings, molecular graphs, or 3D conformer-based features, followed by prediction of solubility, permeability, stability, or binding-related properties. In parallel, retrosynthetic analysis can estimate route feasibility, reaction steps, reagent availability, and synthetic accessibility. Candidates with favorable predicted properties and feasible synthesis routes can then be prioritized for synthesis or procurement, followed by analytical confirmation, physicochemical testing, biological evaluation, or formulation assessment. The resulting experimental data can be returned to the model through active learning or uncertainty-aware retraining. Thus, Figure 1 represents not only a conceptual SPP framework, but also a practical closed-loop strategy for connecting molecular design, synthesis feasibility, experimental validation, and model refinement. The objective of this review is to synthesize current advances in machine-learning-driven molecular design, with a focus on elucidating SPP relationships and enabling the development of synthetically feasible and pharmaceutically relevant compounds.

Accordingly, this review aims to: (i) examine molecular representations that capture both structural and reaction-relevant information; (ii) evaluate machine learning approaches for linking structure with properties and performance; (iii) analyze advances in reaction prediction and retrosynthetic planning, including benchmark-driven progress based on patent-derived reaction datasets; and (iv) identify challenges in achieving interpretable, synthesis-aware, and experimentally validated workflows [12,13].

In response to the rapid evolution of artificial intelligence (AI)-driven molecular design, this review also critically examines transformer-based molecular modeling, molecular foundation models, diffusion-based generation, latent-space optimization, E(3)-equivariant architectures, and joint ligand–protein modeling, with emphasis on their pharmaceutical relevance, limitations, and validation requirements.

Methodologically, this article was designed as a structured narrative review supported by PRISMA-aligned systematic search and screening procedures. This design was selected because the reviewed literature is methodologically heterogeneous, spanning molecular representations, ML architectures, pharmaceutical endpoints, retrosynthetic workflows, generative models, interpretability methods, and validation strategies. Therefore, the objective was not quantitative pooling or meta-analysis, but structured thematic synthesis and critical integration of evidence across diverse computational and pharmaceutical chemistry studies.

## 2. Review Methodology: Structured Narrative Review with PRISMA-Aligned Search Elements

This review was designed as a structured narrative review with PRISMA-aligned systematic search elements. PRISMA 2020 reporting guidance and PRISMA-S search reporting principles were used to improve transparency in database selection, search strategy reporting, duplicate removal, eligibility screening, and study selection [18,19]. However, this manuscript should not be interpreted as a full systematic review with quantitative synthesis or meta-analysis. This distinction is important because the included studies differ substantially in application domain, molecular representation, machine learning architecture, dataset type, validation strategy, performance metric, and pharmaceutical endpoint. Therefore, the evidence was synthesized thematically and critically, rather than statistically pooled.

### 2.1. Databases and Search Strategy

Five databases were selected to ensure comprehensive coverage across biomedical, chemical, and computational domains. PubMed (Available online: https://pubmed.ncbi.nlm.nih.gov, accessed on 11 April 2026) was used for pharmaceutical and clinical research records. Web of Science Core Collection (Available online: https://www.webofscience.com/wos/woscc/basic-search, accessed on 28 December 2025) was used for multidisciplinary indexed literature and citation tracking. IEEE Xplore (Available online: https://ieeexplore.ieee.org, accessed on 12 January 2026) was used for machine learning and algorithm development studies. In addition, Scopus Available online: https://www.scopus.com, accessed on 9 January 2026) was included to provide broad coverage of chemical, pharmaceutical, and interdisciplinary research, including journals not fully indexed in Web of Science. ChemRxiv (Available online: https://chemrxiv.org, accessed on 18 January 2026) was used as a supplementary source to identify recent preprints in chemical sciences, particularly for emerging machine learning approaches in molecular design and synthesis.

The search was conducted from database inception to 22 January 2026. The final query on this date served as the update search prior to submission. The strategy was structured around two concept blocks. The first block targeted pharmaceutical and chemical domains. It included formulation design, molecular design, excipients, synthesis pathways, process engineering, critical quality attributes, dissolution, stability, and drug performance. The second block targeted machine learning. It included artificial intelligence, deep learning, neural networks, support vector machines, random forests, gradient boosting, Bayesian optimization, Gaussian processes, active learning, transfer learning, transformers, and graph neural networks. Term selection was informed by prior studies on machine learning in formulation and molecular design [20,21,22,23]. Search syntax was adapted to each database. PubMed queries used Boolean operators with Title or Abstract fields and Medical Subject Headings (MeSH) expansion where applicable. Web of Science queries used Topic Search fields with Boolean logic. IEEE Xplore queries were adjusted to platform-specific indexing fields. The database-specific search strategies, including formulation-focused queries for biomedical databases and molecular-design-oriented queries for engineering databases, are summarized in Table S1 to ensure transparent and reproducible literature retrieval [18,19,24]. The IEEE Xplore search strategy was tailored to emphasize machine-learning-driven molecular design and generative modeling approaches, reflecting the database’s focus on computational methods rather than formulation-oriented terminology. For Scopus, the search strategy was implemented using TITLE-ABS-KEY fields, referring to title, abstract, and keyword fields, with Boolean operators and keyword combinations aligned with the defined concept blocks. Scopus indexing allowed for integration of chemical, pharmaceutical, and engineering literature within a unified query structure. For ChemRxiv, keyword-based searches were performed using simplified Boolean combinations focused on molecular design, machine learning, and chemical synthesis. As ChemRxiv hosts non-peer-reviewed preprints, records retrieved from this source were screened with additional caution and were included only if they provided substantial methodological detail and clear relevance to pharmaceutical chemistry.

Additional terms included “foundation model,” “Bidirectional Encoder Representations from Transformers (BERT),” “ChemBERTa,” “MolE,” “ChemFM,” “molecular property prediction,” “ADMET foundation model,” “MolMIM,” “BioNeMo,” “diffusion model,” “equivariant diffusion,” “E(3)-equivariant,” “geometric deep learning,” “co-folding,” “protein–ligand foundation model,” “joint structure and affinity prediction,” “Boltz-2,” “uncertainty calibration,” and “prospective validation” [25,26,27,28,29,30,31,32,33,34]. This update was introduced to ensure that this review reflects not only established machine learning methods but also the current frontier of molecular design, ADMET prediction, structure-based drug discovery, and experimentally grounded validation.

Manual screening of reference lists from eligible studies and key review articles was performed to identify additional records not retrieved during database search. Search strategies were adapted to database-specific indexing characteristics, with biomedical databases emphasizing pharmaceutical formulation and drug performance terminology, and engineering databases emphasizing machine-learning-driven molecular design and generative modeling approaches. To ensure completeness, overlap between databases was assessed, and duplicate records across PubMed, Web of Science, Scopus, IEEE Xplore, and ChemRxiv were removed prior to screening. The inclusion of Scopus improved coverage of Elsevier-indexed chemistry journals, while ChemRxiv provided early access to emerging methodologies not yet available in peer-reviewed databases.

**Figure 2:**
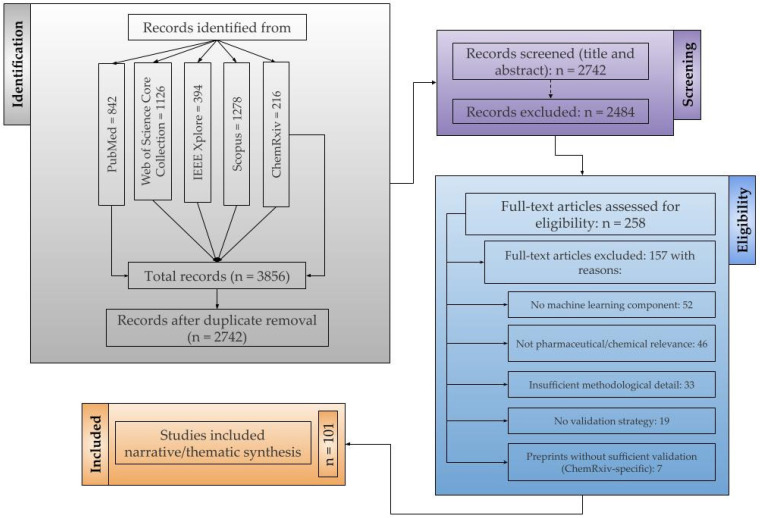
PRISMA 2020 flow diagram of study selection process.

The study selection process is summarized in Figure 2, which presents the PRISMA 2020 flow diagram incorporating records retrieved from PubMed, Web of Science, IEEE Xplore, Scopus, and ChemRxiv, including duplicate removal, screening, eligibility assessment, and final inclusion.

Additional records from ChemRxiv were subjected to stricter eligibility criteria due to their non-peer-reviewed status and were included only when methodological transparency and validation procedures were clearly reported.

### 2.2. Inclusion and Exclusion Criteria

The inclusion and exclusion criteria applied during study selection are summarized in Table 1 to ensure consistent eligibility assessment and methodological transparency. Eligibility criteria were defined before screening to maintain consistency with the study objective. Selection required direct relevance to machine learning applied in chemical or pharmaceutical problems and sufficient methodological transparency.

Studies were included if they addressed a chemical or pharmaceutical task, including molecular design, retrosynthesis, formulation development, excipient selection, process optimization, prediction of physicochemical properties, or drug performance. Eligible studies were required to implement a machine learning or artificial intelligence method for prediction, classification, or optimization. For de novo molecular design studies, priority was given to reports that included synthetic feasibility assessment, retrosynthetic analysis, experimental testing, or prospective validation. Purely generative studies without validation were treated cautiously or excluded where no chemical evaluation was provided. Eligible studies were also required to report descriptors or input representations with explicit definition, including molecular descriptors, fingerprints, graph representations, or sequence-based encodings such as SMILES and SELFIES [35,36]. A defined validation procedure was required, including clear separation of training and test data, cross-validation or external validation, and quantitative performance metrics [18,19]. Studies also had to provide sufficient methodological detail to allow for technical evaluation of model structure, preprocessing, and evaluation design. Peer-reviewed full-text articles in English were included as the main evidence base. ChemRxiv preprints were considered only as supplementary evidence when they provided clear methodological detail, direct relevance to pharmaceutical chemistry, and transparent validation information.

Studies were excluded if they had no direct relevance to chemical or pharmaceutical applications. Studies focused only on inorganic materials, polymers, catalysts, or non-pharmaceutical materials were excluded unless they directly addressed drug-like molecules or pharmaceutical performance. Studies were also excluded if they lacked a machine learning component, used predictive tools without disclosure of model structure, descriptor definition, or preprocessing steps, or did not report a validation protocol or performance metrics. Abstracts, editorials, non-peer-reviewed publications, and studies with insufficient technical detail for extraction of model or data characteristics were excluded. Records retrieved from ChemRxiv or other preprint repositories were screened with additional caution. Preprints without sufficient methodological detail, validation strategy, or clear chemical relevance were excluded. Only preprints with high technical transparency and direct relevance to pharmaceutical machine learning applications were considered. A strict transparency requirement was applied throughout screening. Studies lacking descriptor definition or validation design were excluded. This requirement ensured comparability across studies and supported technical interpretation of reported performance. Overall, the eligibility criteria were designed to ensure that included studies not only demonstrated predictive performance but also maintained chemical relevance, methodological transparency, and experimental or synthesis-aware validation.

### 2.3. Data Extraction Framework

Table 2 presents the structured data extraction framework, which defines the variables used to standardize representation, model configuration, and validation assessment across studies. The extraction protocol was defined before full-text review to support consistent comparison of representation design, model configuration, and validation quality.

The first extraction block focused on molecular representation. Each study was classified according to its encoding strategy, including physicochemical descriptors, fragment-based fingerprints, molecular graphs, and string-based encodings such as SMILES or SELFIES [35,36]. Tokenization details were recorded for sequence-based representations, including vocabulary definition, sequence length handling, padding, truncation, and masking. Representation choice was linked to model compatibility and prediction task. The second extraction block focused on ML models. Model families were recorded for each study and included classical methods such as support vector machines, random forests, and gradient boosting, as well as deep learning methods such as convolutional networks, recurrent networks, transformers, and graph neural networks [20,21,22,23]. Architecture details were extracted when reported, including layer configuration, hidden dimensions, attention mechanisms, optimizer selection, and regularization methods.

Validation design was recorded as a separate field. This included data split strategy, cross-validation scheme, external validation status, and evaluation metrics [1,2]. Classification metrics included accuracy, precision, recall, F1 score, and AUROC. Regression metrics included RMSE, MAE, and R^2^. Additional field captured ablation evidence where available. Studies were assessed for controlled comparison between full models and reduced variants under identical training conditions. This information supports evaluation of component-level contribution. All extracted variables were organized into a standardized evidence matrix, enabling comparison across representation type, model class, validation design, and reporting quality. This structure supported the identification of consistent design patterns and recurring reporting deficiencies in machine learning applications for chemical and pharmaceutical systems.

### 2.4. Analytical Framework for Structure–Property–Performance (SPP) Mapping

A formal analytical scheme was defined to compare studies on the basis of explicit linkages between molecular structure, intermediate physicochemical properties, and downstream performance endpoints. The framework separates evidence into two directional mappings: structure → property and property → performance. This separation enables consistent evaluation across heterogeneous study designs and reporting styles.

*Structure → Property Mapping:* The first mapping evaluates how molecular structure is translated into measurable or predicted physicochemical attributes. For each study, the representation of chemical structure was recorded, including descriptors, fingerprints, graph-based encodings, or sequence-based formats such as SMILES and SELFIES. The analysis then examined whether the selected representation preserved chemically relevant information required for property prediction tasks. Property endpoints extracted in this stage included solubility, lipophilicity, permeability, stability, dissolution rate, and other experimentally measured or computationally estimated attributes. The framework assessed the correspondence between structural features and predicted properties by examining model type, feature importance reporting, and interpretability outputs when available. Studies were categorized according to whether the mapping relied on classical quantitative structure–property relationships or data-driven nonlinear models [1,18,19]. Consistency across studies was evaluated using three criteria. First, descriptor transparency was verified to ensure that structural inputs were explicitly defined. Second, model validity was assessed through reported validation procedures and performance metrics. Third, interpretability evidence was examined to determine whether the relationship between structural features and predicted properties was mechanistically meaningful or purely statistical [20,36,37].

*Property → Performance Mapping:* The second mapping evaluates how predicted or measured properties translate into pharmaceutical performance outcomes. Performance endpoints included bioavailability, dissolution behavior, stability under storage conditions, and in vitro or in vivo drug response. Each study was assessed for the presence of a clear linkage between intermediate properties and final performance metrics. The analysis examined whether property variables were directly used as inputs to predictive models or indirectly linked through sequential modeling stages. Studies were categorized into direct mapping approaches, where properties were used to predict performance in a single model, and multi-stage approaches, where structure → property and property → performance mappings were modeled separately. Comparability across studies was established by examining validation design, metric selection, and consistency of reported endpoints. Regression-based metrics such as RMSE and MAE were used for continuous performance variables, while classification metrics such as accuracy and AUROC were used for categorical outcomes. Preference was given to studies that reported external validation or independent test sets, as these provide stronger evidence for generalization [18,19].

*Cross-Study Integration:* To enable systematic comparison, both mappings were integrated into a unified evidence matrix. Each study was positioned according to its coverage of the two mapping stages and the level of methodological transparency. Studies that explicitly connected structure, property, and performance within a single modeling pipeline were distinguished from those addressing only partial relationships. This framework allows for identification of consistent modeling strategies and recurring limitations. In particular, it highlights whether predictive performance is supported by interpretable structure–property relationships and whether property-level predictions translate into reliable performance outcomes. The approach supports structured synthesis of machine learning applications in pharmaceutical chemistry and facilitates comparison across diverse datasets, model architectures, and experimental settings.

Because of the heterogeneity of study designs, model architectures, datasets, endpoints, and validation metrics, no meta-analysis was performed; instead, the findings were synthesized using structured thematic comparison across representation type, model class, validation approach, and pharmaceutical relevance.

## 3. Molecular Representations in Pharmaceutical Chemistry

Molecular representations provide the interface through which chemical intuition, traditionally expressed in terms of functional groups, stereochemistry, and reactivity, is converted into machine-readable formats suitable for algorithmic processing. Accurate encoding of chemical structure strongly influences model behavior in pharmaceutical ML tasks. Representation choice determines the available feature space, model compatibility, and interpretability. This section categorizes commonly used molecular representations and evaluates their chemical relevance.

### 3.1. 1D and 2D Molecular Descriptors

Molecular descriptors encode chemical information as numerical features derived from structure. Common physicochemical descriptors include partition coefficient (logP), polar surface area (PSA), molecular weight (MW), hydrogen bond donors (HBD), and hydrogen bond acceptors (HBA). These descriptors summarize polarity, size, and intermolecular interaction potential, which are directly linked to absorption, distribution, and solubility behavior [1,2,3]. They are often described as 1D and 2D representations because they summarize molecular composition and topology without explicitly encoding full spatial structure. Descriptor-based approaches are widely used in quantitative structure–property and structure–activity modeling. They enable compatibility with classical machine learning models such as support vector machines and random forests. Descriptor vectors are fixed-length and computationally efficient.

Their main limitation is information compression. Many descriptors rely on predefined formulas and do not encode full structural topology. Subtle stereochemical effects and long-range intramolecular interactions are often lost. Descriptor selection also introduces bias since feature definitions are fixed prior to model training. This restricts the capacity to learn new chemical patterns beyond the encoded feature set [4].

### 3.2. String-Based Representations

String-based representations encode molecules as sequences of characters or tokens. The most widely used formats are SMILES and SELFIES. SMILES represents atoms, bonds, and branching using a compact linear notation. SELFIES uses a grammar-based token system, which supports valid chemical structures during decoding [5,6]. These representations are compatible with sequence-based models, including recurrent neural networks and transformer architectures. Tokenization converts strings into discrete units. Token design affects model performance. Character-level tokenization preserves fine-grained syntax. Substructure-level tokens capture functional groups and recurring motifs [7]. String representations support implicit learning of chemical substructures. Models trained on large corpora identify patterns associated with functional groups, ring systems, and bonding arrangements. This enables end-to-end learning without manual feature engineering. Limitations include dependence on syntax conventions. SMILES permits multiple valid encodings for the same molecule, which introduces variability during training. Sequence length variability affects model stability. SELFIES addresses validity constraints but produces longer sequences, which increases computational cost.

### 3.3. Graph-Based Representations

Graph-based representations model molecules as mathematical graphs. Atoms are treated as nodes. Bonds are treated as edges. Node features encode atomic properties such as element type, valence, and hybridization. Edge features encode bond order and connectivity. This representation preserves full molecular topology. Graph neural networks operate directly on this structure using message passing between nodes. Information propagates along bonds, allowing models to learn local and global structural relationships [8,9]. Graph representations capture connectivity, ring systems, and branching patterns with high fidelity. They support tasks involving reactivity prediction, quantum property estimation, and structure-based optimization. Limitations include computational complexity. Graph models require specialized architectures and are sensitive to graph size and feature design. Long-range interactions require deeper message passing layers, which increases training complexity.

### 3.4. Representation–Chemistry Alignment

Representation choice must match the chemical phenomenon under investigation. Descriptor-based representations are suitable for property prediction tasks where global physicochemical features dominate. These include solubility, permeability, and lipophilicity estimation. String-based representations are appropriate for generative modeling and sequence learning tasks. They support molecular design workflows where new structures are generated and evaluated. Token-based learning captures recurring chemical motifs without predefined feature engineering [7]. Graph-based representations are suited for tasks requiring explicit structural reasoning. These include reaction prediction, binding affinity estimation, and topology-sensitive property modeling. Graph models retain detailed connectivity information, which is essential for mechanistic interpretation [8,9,10]. Beyond graph topology, advanced 3D and quantum-informed representations incorporate electron density distributions, electrostatic potentials, and orbital-level features (e.g., highest occupied molecular orbital–lowest unoccupied molecular orbital (HOMO–LUMO) energies), which are critical for modeling intermolecular interactions and target binding in pharmaceutical systems [38]. No single representation satisfies all requirements. Descriptor methods provide efficiency but lose structural detail. String methods enable flexible learning but depend on encoding rules. Graph methods preserve structure but increase computational cost. Selection must be guided by task definition, data availability, and required level of chemical interpretability. Table 3 compares major molecular representation strategies according to encoded chemical information, machine learning compatibility, interpretability, and suitability for pharmaceutical chemistry tasks.

Table 3 indicates that molecular representation choice defines the chemical information available to machine learning models. Descriptor-based representations provide direct physicochemical meaning and support interpretable property modeling, especially for size, polarity, hydrogen bonding, and permeability-related endpoints [4,39,40]. Fingerprints extend this representation by encoding substructure patterns and atom environments, which supports similarity analysis and QSAR modeling [41]. String-based formats support sequence learning and molecular generation, with SMILES providing compact chemical notation and SELFIES improving validity control during generative modeling [35,36,42]. Graph-based representations preserve atom–bond connectivity and molecular topology, which supports topology-sensitive prediction tasks and message-passing neural models [37,43,44]. Three-dimensional representations extend molecular graphs with coordinates, distances, angles, and conformational information, which improves modeling of stereochemistry, shape, and spatial interactions [45,46,47]. Reaction-based representations encode reactants, products, reagents, and reaction centers, making them suitable for retrosynthesis and reaction prediction [6,48,49]. This comparison supports task-specific representation selection based on endpoint type, dataset size, model class, and required chemical interpretability.

### 3.5. Geometric Deep Learning and Coordinate-Aware Molecular Representations

Geometric deep learning extends molecular machine learning beyond fixed descriptors, molecular strings, and topological 2D graphs by incorporating the spatial geometry of molecules. In pharmaceutical chemistry, this is important because many relevant molecular properties depend not only on atom connectivity, but also on three-dimensional conformation, stereochemistry, intramolecular distances, torsion angles, electrostatic complementarity, and the spatial arrangement of functional groups. These factors influence ligand binding, chiral recognition, pharmacophore matching, membrane permeability, solubility, conformer-dependent reactivity, and protein–ligand interaction geometry [45,46,47]. Conventional 2D graph neural networks represent molecules as atoms connected by bonds. In these models, message passing occurs over molecular topology: atoms exchange information with neighboring atoms through bond-defined edges. This approach preserves connectivity, ring systems, branching patterns, and local chemical environments, but it does not directly encode continuous atomic coordinates unless geometric features are added separately. Therefore, a 2D graph model may distinguish atom–bond arrangements, but it may not fully capture differences between conformers, stereoisomers, binding poses, or spatially separated functional groups that become close in three-dimensional space.

Coordinate-aware geometric models address this limitation by using atomic positions as part of the learning process. Three-dimensional graph neural networks and E(n)-equivariant neural networks are designed so that predictions remain physically consistent when a molecule is translated, rotated, or reflected in space. In this context, equivariance means that if the input coordinates are rotated or translated, the internal coordinate-dependent features transform in the same way, while scalar predictions such as energy, affinity class, or property values remain invariant. This property is important for molecular modeling because the biological or physicochemical meaning of a molecule should not depend on its arbitrary orientation in a coordinate system.

E(n)-equivariant graph neural networks differ from conventional 2D graph neural networks in how they handle geometry. A 2D graph model mainly learns from atom identities, bond types, and topological neighborhoods. By contrast, an equivariant model updates atom features using both connectivity and spatial relationships, such as interatomic distances and coordinate-dependent messages. This enables the model to learn conformational effects, steric constraints, long-range interactions, and protein–ligand contact geometry more directly. Such models are particularly relevant for binding-affinity prediction, molecular docking support, conformer-sensitive property prediction, pharmacophore modeling, and structure-based molecular optimization [45,46,47].

Continuous coordinate-based diffusion models provide a related but distinct approach. Instead of only predicting properties from a given molecular structure, diffusion models can generate or refine molecular geometries by starting from noisy atomic coordinates and progressively denoising them into plausible molecular conformations or ligand poses. In molecular design, this allows the model to learn distributions over three-dimensional structures rather than only over 2D graph connectivity or SMILES strings. Target-conditioned diffusion models can also incorporate protein-binding pocket information, enabling the generation of molecules or conformers that are spatially compatible with a binding site [32,33,34,50].

The distinction between 2D graph models, E(n)-equivariant GNNs, and coordinate-based diffusion models is therefore central to pharmaceutical applications. Topological 2D graphs are useful for many structure–property tasks where connectivity and local substructure patterns dominate. E(n)-equivariant GNNs are more suitable when conformational geometry, stereochemistry, and spatial interactions are important. Continuous coordinate-based diffusion models are especially useful for generative and structure-based workflows because they can sample or refine 3D molecular arrangements under geometric or target-specific constraints. However, coordinate-aware models also introduce additional challenges. Their outputs depend on conformer generation, protonation and tautomeric state assignment, pose selection, energy filtering, and the quality of structural training data. Therefore, geometric deep learning models should be evaluated using multiple conformers, pH-aware molecular-state preparation, external structural or binding datasets, and prospective experimental validation where possible.

### 3.6. Molecular Foundation Models and Frontier Representation Learning

Recent pharmaceutical machine learning has shifted from task-specific models toward molecular foundation models trained on large molecular corpora using self-supervised, weakly supervised, or multi-task objectives. Earlier SMILES-based transformer models demonstrated that molecular strings can be processed in a language-modeling framework, but newer approaches increasingly combine chemical pretraining with ADMET-specific fine-tuning and graph-based molecular representation learning [25,26,27,28]. For example, MolE adapts a transformer architecture to molecular graphs and applies two-stage pretraining, first to learn chemical structure from large-scale molecular graph data and then to incorporate biological information for downstream drug-discovery tasks [25]. Similarly, MolPROP integrates molecular language representations with graph-based molecular information for property prediction, highlighting the growing interest in multimodal foundation-style architectures that combine SMILES-derived representations with molecular graph features [26]. ChemFM further illustrates the scaling of chemical foundation models by using large-scale causal language modeling to support chemical design, molecular property prediction, and reaction-related tasks [27]. SPMM represents a complementary molecular foundation-model direction by enabling bidirectional generation and prediction between molecular structures and property profiles within a unified transformer-based framework [28].

These models differ from classical descriptor-, fingerprint-, or task-specific graph neural network models because they aim to learn transferable chemical representations that can be adapted across multiple downstream endpoints. This is particularly relevant for ADMET prediction, where experimental datasets are often small, noisy, proprietary, or assay-specific. However, the advantages of foundation models are not universal. Reported performance depends strongly on benchmark construction, endpoint definition, molecular split strategy, and chemical overlap between pretraining data and test molecules. Foundation models may also learn dataset-specific shortcuts rather than chemically causal relationships. Therefore, their evaluation should include scaffold-, time-, or cluster-based splits, external validation, uncertainty calibration, and domain-of-applicability analysis. In pharmaceutical chemistry, a foundation model is most useful when its predictions can be connected to interpretable molecular drivers, assay metadata, and prospective experimental confirmation [25,26,27,28].

Therefore, transformer-based and foundation-style molecular models should be evaluated not only by retrospective benchmark performance, but also by their ability to transfer across chemical series, preserve chemically meaningful representations, support ADMET-relevant prediction, and remain reliable under scaffold-, time-, cluster-, and external-validation settings.

## 4. Structure–Property Relationships (SPRs)

Structure–property relationships (SPRs) describe the association between molecular structure and measurable physicochemical behavior. In pharmaceutical chemistry, SPR analysis supports prediction of solubility, lipophilicity, permeability, and related developability endpoints. Machine learning extends classical SPR modeling by capturing nonlinear associations among descriptors, molecular fragments, graph features, and measured properties [51,52,53,54]. Recent studies also show stronger use of graph models, pretrained chemical language models, and explainable methods for molecular property prediction [55,56,57,58]. The machine learning value in SPR analysis depends on chemical plausibility. A predictive model with weak chemical coherence has limited value for molecular design.

### 4.1. Physicochemical Property Prediction

Solubility is a major endpoint in pharmaceutical chemistry. It affects dissolution, absorption, formulation strategy, and dose feasibility. Molecular size, polarity, ionization state, hydrogen-bonding capacity, aromaticity, crystal packing, and conformational flexibility all influence aqueous solubility. Descriptor-based models often use molecular weight, logP, topological polar surface area, hydrogen-bond donors, hydrogen-bond acceptors, rotatable bonds, and charge-related features to estimate solubility. Recent comparative work confirms the value of curated descriptors and machine learning models for pharmaceutical aqueous solubility prediction [51,52,59]. Recent solubility studies also integrate molecular graphs, electrostatic potential features, Mordred descriptors, and ensemble machine learning models to improve prediction and interpretation [59]. Lipophilicity is usually represented by logP or logD. It influences membrane partitioning, permeability, protein binding, metabolic liability, and tissue distribution. Excessive lipophilicity often increases nonspecific binding and developability risk. Low lipophilicity limits passive diffusion for many drug-like molecules. Machine learning models estimate lipophilicity using descriptors, fingerprints, graph features, and fragment-level encodings related to hydrophobic surface area, heteroatom distribution, aromaticity, and ionizable groups [53,54].

Permeability reflects the ability of a molecule to cross biological barriers. It is influenced by molecular size, polarity, hydrogen bonding, ionization, conformational flexibility, and intramolecular hydrogen bonding. Permeability prediction is more complex than isolated solubility or lipophilicity prediction because assay format, membrane type, transporter effects, and pH-dependent ionization can influence the measured value [60,61]. Machine learning models are most informative when structural variables are paired with assay metadata and external validation.

### 4.2. Chemical Determinants of Properties

Functional groups influence molecular properties through polarity, ionization, hydrogen bonding, reactivity, and intermolecular interactions. Carboxylic acids, amines, amides, alcohols, sulfonamides, nitriles, halogens, and heteroaromatic systems alter solubility, lipophilicity, and permeability in different ways. Their effects depend on the local molecular environment, neighboring substituents, and charge state. Electronic effects shape pK_a_, dipole moment, hydrogen-bond strength, aromatic electronics, and metabolic stability. Electron-withdrawing and electron-donating substituents influence polarity and ionization, which then affect solubility and membrane partitioning. These effects also modify binding and reactivity through charge distribution and orbital characteristics. Steric effects influence molecular shape, conformational restriction, crystal packing, and exposure of polar atoms. Bulky substituents alter target binding and metabolic stability. They also influence solubility through hydrophobic surface area and lattice interactions. Rotatable bonds and conformational flexibility affect entropy, membrane crossing, and availability of hydrogen-bonding sites. A chemically meaningful SPR model should reflect these determinants. Feature ranking, fragment attribution, or atom-level interpretation should be consistent with known effects of polarity, ionization, hydrophobicity, hydrogen bonding, electronic substitution, and steric bulk. Models with high numerical accuracy but chemically implausible explanations require cautious use in optimization.

Table 4 summarizes the major physicochemical properties, their chemical determinants, common descriptor classes, suitable machine learning models, and interpretation methods used in SPR analysis.

The table indicates that chemically meaningful SPR interpretation requires agreement between model-derived importance patterns and established effects of polarity, ionization, hydrophobicity, hydrogen bonding, electronic substitution, and steric demand.

### 4.3. ML Interpretation of SPRs

Interpretability methods connect model outputs with chemical reasoning. Feature-importance and attribution-based approaches identify molecular descriptors, fragments, atoms, or substructures that strongly influence model predictions. Recent explainable artificial intelligence (XAI) studies in drug discovery emphasize that interpretable outputs are most chemically useful when highlighted features correspond to recognizable molecular properties or structural motifs, rather than abstract numerical signals [62,63]. In descriptor-based SPR/QSPR (quantitative structure–property relationship) models, highly ranked variables may include logP, polar surface area, molecular weight, hydrogen-bonding descriptors, charge-related descriptors, and fragment counts. These outputs are especially informative when the selected features have direct chemical meaning and can therefore be related to physicochemical behavior, molecular recognition, toxicity risk, or property optimization [62,63].

SHAP analysis provides both local and global feature-contribution estimates. It supports compound-level interpretation by identifying which descriptors increase or decrease predicted solubility, lipophilicity, permeability, or broader ADME-related properties [64,65]. Recent explainable molecular modeling studies have used SHAP to identify descriptor contributions across drug-profile predictions and to improve transparency in model interpretation [65]. Attention mechanisms and atom-level attribution provide additional interpretation routes for sequence- and graph-based models. In SMILES-based transformer models, attention patterns may highlight tokens associated with functional groups, ring systems, or reaction centers. In graph models, atom- or bond-level attribution can identify substructures contributing to predicted properties, while recent graph neural architectures further demonstrate the importance of topology-aware learning for molecular property prediction [56,57,58,66]. However, attention scores should not be interpreted as direct mechanistic proof. They require comparison with analogue-series behavior, substituent effects, and experimental observations.

Overall, SPR models are most useful when they identify chemically plausible structural determinants of physicochemical behavior, such as polarity, ionization, hydrogen bonding, lipophilicity, steric effects, and conformational flexibility. These outputs provide the property-level basis for the PPR and integrated SPP analyses discussed in the following sections.

## 5. Property–Performance Relationships (PPRs)

Property–performance relationships (PPRs) connect measurable molecular and formulation properties with pharmaceutical performance endpoints. In pharmaceutical chemistry, PPR analysis links solubility, lipophilicity, permeability, particle attributes, excipient interactions, and degradation risk with dissolution, stability, systemic exposure, and therapeutic reliability. Machine learning supports PPR modeling by integrating molecular descriptors, formulation variables, process parameters, and experimental performance data into predictive models. Recent pharmaceutical studies show increased use of machine learning for dissolution profile prediction, solubility-linked bioavailability assessment, stability modeling, and drug development decision support [59,67].

### 5.1. Dissolution Behavior

Dissolution behavior is a primary performance endpoint for oral drug products. It depends on the intrinsic solubility of the active pharmaceutical ingredient, particle size, surface area, wettability, solid-state form, excipient composition, tablet porosity, and manufacturing conditions. Poor aqueous solubility often reduces dissolution rate and limits absorption, especially for compounds with high permeability but low solubility [58,59]. Recent solubility modeling work confirms that aqueous solubility directly influences bioavailability and therapeutic outcomes, and that molecular graphs, electrostatic potential maps, and tabular descriptors improve solubility prediction when evaluated against curated datasets [59]. Dissolution also depends on particle-level and formulation-level interactions. Particle aggregation, hydrophobic surfaces, polymer swelling, matrix erosion, disintegration, and active pharmaceutical ingredient (API)–excipient compatibility affect the release profile. Machine learning models are useful when dissolution performance is controlled by multiple variables rather than a single physicochemical property. A 2025 study using 377 direct-compression formulations showed that RF and XGBoost predicted dynamic release profiles across 11 time points, with fivefold cross-validation R^2^ values of 0.635 ± 0.047 and 0.601 ± 0.091, respectively. The same study also modeled kinetic parameters for release equations, which improved interpretability of predicted dissolution profiles [67].

### 5.2. Stability and Degradation

Stability links chemical structure, formulation environment, storage conditions, packaging, and degradation pathways. Molecular features such as labile ester, amide, lactam, aldehyde, peroxide-sensitive, and photoreactive groups influence degradation risk. Hydrolysis, oxidation, photolysis, isomerization, racemization, and Maillard-type reactions are common pathways in drug substances and drug products. These processes depend on pH, moisture, oxygen, light, temperature, trace metals, excipient impurities, and solid-state mobility. Chemical structure contributes to degradation by defining reactive sites and local electronic environments. Electron-rich aromatic systems may increase oxidative sensitivity. Hydrolytically labile bonds may degrade faster under acidic or basic stress. Steric protection may reduce access of water or nucleophiles to reactive centers. Salt form, polymorphic state, amorphous content, and excipient microenvironment further modify degradation kinetics.

Recent stability modeling work has moved toward combined empirical and data-driven approaches. A 2025 study compared an acceleration factor model with a constrained neural network for tablet dissolution slowdown during storage. The authors linked dissolution change to humidity-extended Arrhenius behavior and first-order relaxation or equilibration processes. The constrained neural network and acceleration factor approach predicted dissolution profiles in packaged tablets and identified boundary conditions related to humidity control [68]. Regulatory guidance also emphasizes stress testing and accelerated stability data for defining degradation pathways, intrinsic stability, and stability-indicating critical quality attributes [69].

### 5.3. Bioavailability

Bioavailability reflects the fraction of administered drug reaching systemic circulation. It depends on dissolution, solubility, gastrointestinal stability, permeability, first-pass metabolism, transporter effects, formulation design, and food effects. The structure → permeability → systemic exposure link is central for many small molecules. Molecular size, polarity, hydrogen-bonding capacity, ionization state, lipophilicity, and conformational flexibility influence passive permeability and absorption potential.

Structure alone rarely explains bioavailability. A compound with favorable permeability may still show poor exposure due to low solubility or slow dissolution. A soluble compound may show low exposure due to poor membrane permeability, degradation in the gastrointestinal tract, efflux transport, or high first-pass metabolism. PPR modeling must connect molecular properties with formulation behavior and biological barriers.

Recent discussions of bioavailability limitations emphasize the combined effects of physicochemical and biological factors, including solubility, permeability, metabolism, transporters, and formulation strategy [70]. Machine learning adds value when it integrates these factors into multi-input prediction models. For absorption-related prediction, food-effect modeling is also relevant because food may alter dissolution, gastric emptying, bile salt solubilization, and intestinal absorption. Recent work shows that machine learning is being applied to food-effect prediction during drug absorption assessment [71].

### 5.4. ML Prediction of Performance

Pharmaceutical performance is multifactorial. Dissolution, stability, and bioavailability depend on molecular structure, formulation composition, process history, storage environment, and biological conditions. Machine learning models are suitable for PPR analysis when these variables interact in nonlinear ways. Tree-based models, ensemble learning, neural networks, graph models, and hybrid mechanistic–ML approaches each offer different advantages depending on endpoint type, dataset size, and interpretability requirements. For dissolution prediction, ML models should incorporate API properties, excipient identities, formulation ratios, process parameters, tablet physical attributes, medium composition, pH, and time-resolved release data. For stability prediction, useful inputs include reactive structural motifs, solid-state properties, water activity, packaging material, humidity, temperature, oxygen exposure, and degradation rate constants. For bioavailability prediction, models should combine solubility, dissolution, permeability, pK_a_, logD, metabolic stability, transporter liability, and formulation descriptors.

Recent evidence supports this multifactorial direction. ML models trained on formulation and dissolution data have predicted full release profiles and kinetic parameters, while solubility models trained on multiple molecular representations have improved aqueous solubility estimation across curated datasets [67]. Regulatory horizon scanning also identifies AI and ML applications across drug discovery, non-clinical development, pharmacokinetics, manufacturing, and broader medicines lifecycle activities, while noting data quality and model interpretability as major constraints [69]. ML-based PPR models should therefore be evaluated according to their ability to connect measurable molecular, formulation, and process variables with performance-relevant outcomes. For dissolution, stability, and bioavailability, useful models should identify the limiting property or formulation factor that controls performance, rather than treating performance as an isolated numerical endpoint. Table 5 summarizes these relationships and highlights how different pharmaceutical endpoints require different input variables, model types, and interpretation strategies within a chemically informed PPR framework.

## 6. Integrated Structure–Property–Performance (SPP) Mapping

Building on the SPR and PPR layers discussed above, integrated structure–property–performance mapping links molecular structure, intermediate properties, formulation variables, and final pharmaceutical outcomes within a single decision-support workflow. Its purpose is not to repeat isolated property or performance prediction, but to connect these levels so that molecular design, synthetic feasibility, formulation behavior, and developability assessment can be evaluated together. Recent work in molecular property prediction and pharmaceutical AI shows increasing use of end-to-end learning, multimodal data integration, and model interpretation across the medicines lifecycle [69,72,73].

### 6.1. End-to-End ML Pipelines

End-to-end ML pipelines use molecular inputs to predict downstream pharmaceutical behavior. The simplest pipeline begins with molecular structure and generates descriptors, fingerprints, strings, or graph-based representations. These inputs are then passed to an ML model for prediction of physicochemical properties, formulation-relevant attributes, or performance outcomes. In an SPP setting, the prediction target is not limited to solubility, permeability, or stability. It also includes dissolution behavior, degradation risk, bioavailability, and drug product performance. A direct molecular input → performance prediction pipeline is attractive because it reduces manual feature engineering and supports rapid candidate ranking. Graph neural networks, transformers, and pretrained molecular models are increasingly used for this purpose, especially when the input contains structural, sequence, or graph-level information [58]. Recent molecular property prediction studies show continued progress in low-data learning, pretrained chemical models, and adaptive transfer across related tasks [72,73].

A limitation remains. Direct end-to-end prediction may obscure the intermediate chemical logic connecting structure to property and property to performance. For pharmaceutical chemistry, this intermediate logic is important. A model that predicts dissolution or exposure from molecular structure should still show whether the result is driven by solubility, polarity, ionization, permeability, stability, or formulation-sensitive variables. For this reason, end-to-end SPP models should be paired with interpretable intermediate outputs where possible.

### 6.2. Multimodal Data Integration

SPP mapping often requires more than molecular structure. Performance endpoints depend on molecular properties, solid-state form, particle attributes, excipients, processing conditions, storage environment, and experimental assay design. Multimodal integration combines these data streams into a unified prediction model. The molecular data layer includes descriptors, fingerprints, SMILES, SELFIES, molecular graphs, and three-dimensional features. The process layer includes granulation parameters, compression force, coating conditions, drying temperature, mixing time, particle size, and process analytical technology signals. The experimental layer includes solubility, dissolution profiles, permeability data, impurity trends, degradation rates, assay conditions, and pharmacokinetic measurements.

Multimodal SPP models are most useful when the performance endpoint arises from interacting variables. Dissolution is influenced by molecular solubility, crystal form, particle size, tablet porosity, and excipient behavior. Stability is influenced by chemical liability, moisture, temperature, oxygen, packaging, and excipient impurities. Bioavailability is influenced by dissolution, permeability, metabolism, transporter effects, and formulation strategy. Recent AI and ML reviews in pharmaceutical formulation and drug delivery emphasize the value of integrating formulation variables, process parameters, and experimental outputs for performance prediction and product design [74,75]. Multimodal integration also creates methodological risks. Data sources often differ in scale, measurement quality, endpoint definition, and missingness. Molecular datasets may contain thousands of structures, whereas formulation datasets may contain fewer experimental batches. Experimental data may be assay-specific and difficult to compare across laboratories. These issues require careful preprocessing, feature alignment, validation design, and uncertainty analysis.

A practical multimodal SPP model must also define how different input types are fused. In pharmaceutical chemistry, the main fusion paradigms are early fusion, intermediate fusion, and late fusion. Early fusion combines all available variables into a single feature matrix before model training. For example, molecular descriptors or fingerprints can be concatenated with formulation variables, excipient ratios, compression force, particle size, pH, humidity, temperature, and release-time variables. This approach is simple and interpretable, but it is less suitable when molecular inputs are graphs, SMILES/SELFIES strings, or 3D conformers that require specialized encoders.

Intermediate fusion combines learned representations from separate modality-specific encoders. In this design, a graph neural network may encode molecular topology, a transformer may encode SMILES or SELFIES strings, and a multilayer perceptron may encode tabular formulation or process variables. The resulting embeddings are then merged in a shared hidden layer for downstream prediction. This approach is particularly useful for SPP modeling because it allows chemically rich molecular representations to be integrated with process and experimental variables without reducing all inputs to manually defined descriptors. Intermediate fusion is therefore well suited for predicting dissolution profiles, degradation risk, bioavailability-related behavior, or multi-endpoint pharmaceutical performance.

Late fusion combines outputs from independently trained models. For example, one model may predict molecular solubility or permeability from graph/string inputs, another model may predict formulation performance from process variables, and a final meta-model or weighted ensemble may combine these outputs into an overall performance prediction. Late fusion is useful when data sources differ in scale, quality, or availability, or when models are developed separately for molecular, formulation, and experimental domains. However, it may provide weaker mechanistic integration than intermediate fusion because interactions between molecular structure and process variables are learned only at the decision level.

These fusion strategies are especially relevant when tabular process variables must be considered jointly with graph- or string-based molecular inputs. In tablet dissolution prediction, for instance, molecular graph or SMILES embeddings of the active pharmaceutical ingredient can be integrated with excipient ratios, compression force, tablet porosity, dissolution medium, pH, and release-time data. In stability prediction, molecular structural alerts can be combined with moisture, oxygen exposure, packaging material, storage humidity, temperature, and impurity profiles. Thus, multimodal fusion supports the central goal of SPP modeling: connecting molecular structure, formulation/process context, and experimentally observed performance within a unified predictive framework.

To further clarify these fusion paradigms, Table 6 summarizes the main multimodal fusion strategies for SPP modeling in pharmaceutical chemistry. The table compares early, intermediate, and late fusion according to their core concept, input structure, main advantages, limitations, and pharmaceutical applications. This comparison shows that early fusion is most suitable for curated tabular datasets, including formulation and process variables; intermediate fusion is more appropriate when molecular graph, SMILES, or SELFIES embeddings must be combined with formulation and process variables; and late fusion is useful when separate molecular, formulation, or experimental models are developed independently and combined at the decision level [21,23,26,67,74,75].

### 6.3. Cross-Study Comparison Challenges

Cross-study comparison is a major limitation in SPP research. Dataset heterogeneity is common. Studies use different chemical spaces, descriptor sets, assay protocols, formulation compositions, manufacturing conditions, and performance endpoints. A model trained on one dataset may not generalize to another dataset if the chemical distribution, experimental protocol, or product type changes. Benchmark inconsistency is another major concern. Molecular property prediction studies often differ in dataset splitting strategy, metric selection, external validation, and baseline model selection. Random splits may overestimate performance when structurally similar molecules occur in training and test sets. Scaffold splits and time splits provide stricter tests but are not used consistently across studies. Recent benchmark-focused molecular prediction studies continue to highlight dataset splitting and evaluation design as central factors in model comparability [55,76]. Endpoint inconsistency also affects interpretation. Solubility may be reported under different pH, temperature, salt form, or assay conditions. Permeability may be measured using different cell lines, artificial membranes, or intestinal models. Dissolution may vary by medium, apparatus, agitation rate, and sampling schedule. Stability may depend on packaging, humidity, temperature, and analytical method. These variations reduce direct comparability across studies. SPP models should therefore be assessed using more than internal accuracy. Stronger evidence comes from external validation, clearly defined endpoints, transparent preprocessing, chemically meaningful interpretation, and reporting of data boundaries. Regulatory-facing discussions of AI and ML in medicines development also identify data quality, representativeness, transparency, and validation as important constraints for reliable use across the medicines lifecycle [69].

**Figure 3:**
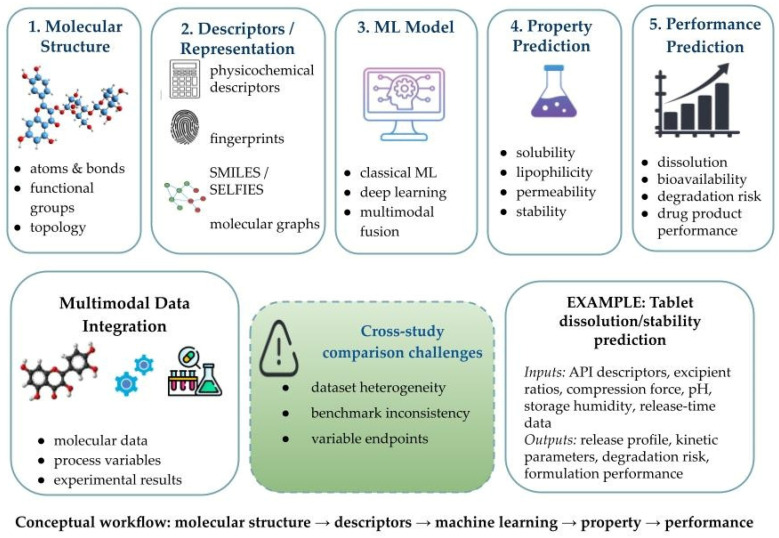
Integrated SPP workflow for pharmaceutical ML, connecting molecular representations, property prediction, multimodal data integration, performance endpoints, and cross-study validation challenges.

Figure 3 illustrates the integrated SPP workflow, linking molecular structure, descriptors or representations, machine learning models, property prediction, and performance prediction. It also highlights the role of multimodal data integration and the main obstacles to cross-study comparison. A representative application of this workflow is tablet dissolution or stability prediction, where molecular descriptors of the API are combined with excipient identity, formulation ratios, process parameters, tablet physical attributes, dissolution media, storage humidity, temperature, and time-resolved experimental measurements. In such a case, the ML model does not only predict an isolated property but connects molecular and formulation-level variables with performance endpoints such as release profile, degradation risk, or bioavailability-related behavior. Similarly, in molecular design, the same workflow can integrate molecular graphs or SMILES-based representations with predicted solubility, permeability, ADMET properties, and experimental assay results. These examples clarify that Figure 3 is intended as a practical SPP mapping template for multimodal pharmaceutical datasets rather than only a generic conceptual pipeline.

### 6.4. Generative Molecular Design and Chemical Foundation Models

Generative molecular design is a core component of AI-assisted pharmaceutical chemistry because it enables the exploration, reconstruction, and optimization of chemical space beyond the screening of existing compound libraries. In the context of the structure–property–performance framework, generative models can be used to propose molecular structures that satisfy predefined property and performance requirements, such as potency, solubility, permeability, metabolic stability, low toxicity, synthetic accessibility, and formulation suitability. This workflow generally involves three connected processes: molecular assimilation, in which chemical structures are encoded into machine-readable representations; latent or distributional learning, in which the model learns structural and property-related patterns from molecular datasets; and molecular reconstitution, in which new candidate structures are decoded, sampled, optimized, or refined [7,31,50,77,78,79,80].

Several model families support this generative workflow. Variational autoencoders encode molecules into continuous latent spaces and generate new structures by sampling and decoding latent vectors [79]. Generative adversarial networks use a generator–discriminator framework to produce molecules that resemble the training distribution, although training instability and mode collapse remain important limitations [80]. Diffusion models generate molecules through iterative denoising and are increasingly important for three-dimensional molecular generation, conformer generation, target-conditioned molecular design, and structure-based drug discovery [32,33,34,50]. Flow-based models provide invertible mappings between molecular structures and latent variables, enabling controlled sampling and likelihood-based generation. Reinforcement-learning optimization is widely used to guide molecular generation toward reward functions that include potency, selectivity, ADMET properties, synthetic accessibility, and drug-likeness [31,78]. Genetic algorithms and evolutionary search methods provide complementary optimization strategies by mutating, recombining, and selecting candidate structures according to predefined fitness criteria. More recent structure-constrained approaches, such as PURE, further attempt to reduce metric bias and improve chemical realism by combining policy-guided reinforcement learning with molecular transformation rules and self-supervised representation learning [81].

Molecular representation strongly affects the quality and validity of generated structures. SMILES strings are widely used because they allow molecules to be processed as sequences by recurrent neural networks, transformers, and chemical language models [35]. However, generated SMILES strings may be syntactically invalid or chemically unrealistic if decoding is not constrained. SELFIES addresses part of this limitation by using a robust grammar in which generated sequences are more likely to correspond to valid molecular structures [36,42]. Graph-based and three-dimensional representations further extend generative design by preserving atom–bond connectivity, stereochemistry, conformational geometry, and binding-site information [37,43,44,45,46,47]. These representations are especially relevant when the design objective depends on spatial complementarity, ligand conformation, or protein–ligand interaction geometry.

Chemical foundation models extend generative molecular design by learning transferable molecular representations from large unlabeled or weakly labeled chemical datasets. Transformer-based models such as ChemBERTa, ChemBERTa-3, and MoLFORMER are trained on molecular strings using self-supervised learning objectives, allowing them to capture chemical syntax, substructural regularities, and structure–property patterns that can be transferred to downstream tasks [82,83]. MoLFORMER demonstrates how large-scale SMILES-based pretraining can generate molecular embeddings useful for property prediction and chemical representation learning [82]. ChemBERTa-3 further illustrates the movement toward reproducible, open-source chemical foundation-model training and benchmarking frameworks [83]. These models can support property prediction, similarity search, virtual screening, ADMET modeling, and generative design when combined with latent-space optimization, reinforcement learning, or diffusion-based generation [25,26,27,28,31,32,33,34,82,83].

Generative large language models are also increasingly used in chemical text mining and molecular design. In chemical text mining, LLMs can assist in extracting compound names, targets, assay results, synthesis conditions, adverse effects, and structure–activity relationship information from the literature. In molecular design, LLM-based systems can support prompt-guided molecule generation, molecule-to-text explanation, text-to-molecule translation, retrosynthetic suggestion, and iterative hypothesis generation [84,85]. Recent studies show that LLMs can read, write, and modify molecular strings according to natural-language prompts, while broader chemistry-agent frameworks connect LLMs with tools for synthesis planning, database search, literature mining, and automated experimentation [84,85]. However, chemically fluent text or valid molecular strings do not necessarily guarantee synthetic feasibility, biological activity, selectivity, safety, or experimental reproducibility. Therefore, LLM-guided molecular design should be combined with chemical constraints, retrosynthetic filtering, ADMET prediction, uncertainty estimation, and prospective experimental validation [77,81,84,85,86]. To clarify the methodological differences among these approaches, Table 7 summarizes the major generative and foundation-model strategies used in molecular design, including VAEs, GANs, diffusion models, flow-based models, reinforcement learning, genetic algorithms, ChemBERTa/MoLFORMER-type chemical foundation models, and generative LLMs. The table compares their core capabilities, main uses in molecular design, key limitations, and recommended validation strategies. This comparison highlights that generative molecular design should not be evaluated only by novelty, validity, or retrospective benchmark performance. Instead, generated candidates should also be assessed for synthetic accessibility, scaffold diversity, ADMET behavior, molecular-state plausibility, uncertainty, and prospective experimental confirmation.

Overall, generative molecular design should be interpreted as a candidate-prioritization and hypothesis-generation approach rather than a standalone discovery solution. Its pharmaceutical value depends on whether generated molecules are valid, novel, diverse, synthetically accessible, chemically stable, biologically relevant, and experimentally testable. Within the SPP framework, the most useful generative workflows are those that connect molecular generation with property prediction, performance evaluation, synthesis feasibility assessment, uncertainty-aware prioritization, and feedback from prospective experimental testing [31,32,33,34,77,81,84,85,86].

### 6.5. Joint Ligand–Protein Modeling and Co-Folding in Structure-Based Drug Discovery

Recent AI-driven molecular design is increasingly moving from ligand-only property prediction toward target-aware modeling, in which molecular generation, binding-pose prediction, protein–ligand interaction modeling, and affinity estimation are integrated into a single computational workflow.

A major frontier in pharmaceutical machine learning is the transition from ligand-only prediction toward joint modeling of ligands, protein targets, binding poses, and binding affinity. Co-folding and biomolecular foundation models attempt to predict protein–ligand complex structures and affinity-related outputs within a unified framework. This development is important because pharmaceutical performance is not determined by ligand properties alone; it also depends on target conformation, binding-site flexibility, water networks, induced fit, protonation states, and assay context. Boltz-2 represents this frontier by extending biomolecular structure prediction toward binding-affinity prediction and by combining co-folding capability with affinity-oriented modeling [29]. Recent biomolecular interaction models, including AlphaFold 3 and Boltz-2-like co-folding approaches, indicate that joint modeling of proteins, ligands, nucleic acids, ions, and modified residues can support early structure-based hypothesis generation, but affinity prediction and lead optimization still require benchmarking against experimental data and physics-based methods [29,30].

Joint ligand–protein models can accelerate early-stage virtual screening, pose hypothesis generation, and target-aware molecular design. Nevertheless, they should not be treated as replacements for experimental binding data, molecular dynamics, or free-energy calculations. Their predictions remain sensitive to protein conformational state, ligand tautomeric and protonation state, crystallographic bias, training-set overlap, and endpoint heterogeneity. For lead optimization, co-folding and affinity-prediction models should therefore be combined with docking, molecular dynamics, free-energy calculations, medicinal chemistry interpretation, uncertainty estimation, and prospective biochemical validation [29,30].

The frontier models discussed above differ not only in their architectures, but also in the pharmaceutical questions they address, the chemical information they encode, and the validation strategies required for reliable application. To provide a more critical comparison of recent AI-driven molecular design approaches, Table 8 summarizes key frontier machine learning frameworks, including molecular foundation models, latent-space generative models, diffusion and E(3)-equivariant models, co-folding approaches, and uncertainty-aware prospective workflows, according to their main capabilities, limitations, and validation needs.

Table 8 shows that frontier pharmaceutical ML frameworks should not be evaluated only by retrospective accuracy or generative novelty. Foundation models require chemically meaningful data splits and external ADMET testing; latent-space and diffusion-based generators require synthetic and experimental feasibility checks; co-folding models require orthogonal structure and affinity validation; and uncertainty-aware workflows require calibration and prospective testing [82,83]. This comparison supports a more critical interpretation of recent ML advances and helps distinguish practical pharmaceutical utility from benchmark-driven performance.

## 7. Chemical Interpretability and Mechanistic Insight

After SPRs, PPRs, and SPP relationships are modeled, interpretability determines whether the resulting ML predictions can be understood in chemically meaningful terms. In this review, interpretability is treated not as a separate modeling goal, but as a requirement for connecting predicted outcomes with recognizable structural features, physicochemical drivers, and experimentally testable hypotheses.

### 7.1. Why Interpretability Matters in Chemistry

Interpretability is required for scientific trust, especially when machine learning outputs are used to guide molecular design, drug discovery, and pharmaceutical decision-making [87,88]. Chemical design decisions depend on whether a prediction has a plausible structural basis. A solubility model should identify polarity, ionization, hydrogen bonding, and crystal-related features as relevant contributors. A stability model should identify labile bonds, electron-rich regions, moisture sensitivity, and oxidative or hydrolytic liabilities. A permeability model should reflect polarity, molecular size, ionization, hydrogen bonding, and conformational flexibility. Interpretability is also relevant for regulatory confidence. AI and ML systems used across the medicines lifecycle raise concerns related to data quality, model transparency, validation, and traceability. Regulatory discussions increasingly emphasize the need to understand model behavior, define the intended use, control data bias, and document model performance across relevant conditions [69,89]. In this setting, chemically interpretable ML supports auditability and reduces the risk of using statistically accurate but chemically implausible predictions.

Scientific interpretation is especially important in low-data and extrapolative settings. Pharmaceutical datasets often contain limited chemical series, assay-specific endpoints, and biased sampling. A model trained under these conditions may learn dataset artifacts rather than causal chemical drivers. Interpretation methods help identify whether predictions are driven by meaningful molecular features or by spurious correlations.

### 7.2. Interpretable ML Techniques

SHAP analysis assigns feature-level contribution values to model predictions. It is widely used for tabular descriptor models and tree-based models. In pharmaceutical chemistry, SHAP supports interpretation of descriptor effects on solubility, lipophilicity, permeability, toxicity, and broader drug-profile endpoints. Recent studies applied SHAP to drug-profile prediction and showed how descriptor contributions can be compared across compounds and endpoints [65]. Feature attribution methods identify the input variables, atoms, bonds, fragments, or substructures that influence a prediction. In descriptor-based models, attribution may highlight logP, polar surface area, molecular weight, hydrogen-bond donors, hydrogen-bond acceptors, rotatable bonds, or charge descriptors. In graph models, attribution may highlight atoms, bonds, ring systems, heteroatoms, or functional groups. Recent graph-based work has moved toward chemistry-aware explanation, including substructure-level masking and concept-based interpretation [90,91]. Attention maps provide another route for interpretation in sequence and graph models. In SMILES-based or reaction-based transformers, attention can indicate tokens linked to reactive centers, functional groups, or chemically informative fragments. Attention patterns should be treated with caution. They are useful diagnostic signals, but they are not direct proof of mechanism. Chemical validation against analogue series, substituent effects, and experimental data remains necessary. Transformer-based chemistry models support reaction prediction and molecular property tasks, but their explanations require domain-level verification [48,55].

Self-interpretable architectures offer a stronger route than post hoc explanations in some cases. Concept-based graph neural networks and chemically constrained architectures can embed interpretable molecular concepts directly into the model structure. Recent work on self-interpretable graph neural networks demonstrates that molecular concepts and structural regions can be incorporated into prediction models to improve transparency in molecular property prediction [90].

Despite their practical value, attribution-based explanations have important limitations. Feature importance, SHAP values, atom attribution, and bond attribution describe how a trained model uses its inputs, but they do not by themselves establish causal chemical mechanisms. Their outputs may change with the selected descriptor set, molecular representation, background distribution, correlated variables, perturbation strategy, random seed, or model architecture. In graph and sequence models, attribution maps may highlight atoms, bonds, or tokens that are statistically useful for prediction without confirming that these features are experimentally responsible for the observed property or activity. Therefore, attribution results should be interpreted as model-behavior diagnostics and should be checked against analogue-series trends, substituent effects, external validation, and experimental evidence.

Table 9 shows that interpretability methods differ in chemical resolution. Descriptor-based explanations support property-level reasoning, whereas graph and attention-based methods support atom-, bond-, or token-level interpretation.

### 7.3. Linking ML Outputs to Chemical Reasoning

ML outputs should be interpreted through known chemical principles. For solubility, an interpretable model should associate increased polarity, ionization, and hydrogen-bonding capacity with improved aqueous interaction, subject to the opposing effects of crystal packing and molecular size. If SHAP or feature importance assigns high relevance to polar surface area, hydrogen-bond donors, ionizable groups, or logD, the explanation is chemically plausible.

For permeability, the interpretation should reflect the balance between membrane partitioning and desolvation cost. Excessive polarity, high hydrogen-bonding capacity, and large molecular size often reduce passive permeability. Moderate lipophilicity and intramolecular hydrogen bonding may improve membrane crossing for selected compounds. A useful model should identify this balance rather than ranking one descriptor in isolation.

For stability, interpretation should identify structural alerts and environmental sensitivities. Aromaticity may improve chemical stability in some systems by reducing conformational flexibility or protecting reactive centers. It may also increase oxidative risk in electron-rich aromatic systems. Amides, esters, lactams, aldehydes, phenols, and peroxide-sensitive motifs may be linked with hydrolysis, oxidation, or photochemical degradation. ML explanations should be checked against expected degradation pathways.

For molecular optimization, interpretability supports actionable design. If a model predicts poor solubility due to high lipophilicity and low polar surface area, the chemist can consider heteroatom insertion, ionizable group tuning, or reduction in hydrophobic aromatic surface. If a model predicts degradation risk due to a labile ester, bioisosteric replacement or steric shielding may be considered. These design actions require the model explanation to map onto chemical reasoning.

### 7.4. Limitations of Black-Box Models

Black-box models create several risks in pharmaceutical chemistry, including limited transparency, weak mechanistic traceability, and reduced confidence in model-guided drug design decisions [87,88]. They may show high internal accuracy but poor external validity. They may depend on dataset-specific artifacts, hidden assay bias, or scaffold similarity between training and test sets. They may also assign importance to features that lack chemical relevance. These risks are amplified when random splits are used in chemically clustered datasets. Post hoc explanations also have important limitations. SHAP values and feature-importance rankings depend on the input representation, background distribution, correlated descriptors, and model structure. Atom- and bond-level attribution can vary according to the perturbation method, graph construction, featurization scheme, and random seed. Attention weights in transformer or graph-attention models may identify tokens, atoms, or substructures that influence model output, but they should not be interpreted as direct evidence of causal chemical importance. Different explanation methods may also produce different rationales for the same prediction. For this reason, explanation results should be interpreted as evidence of model behavior rather than standalone mechanistic proof.

Chemical interpretability requires triangulation. A credible interpretation should align with known physicochemical principles, analogue-series trends, external validation, and experimental observations. Model explanations should be reported with their assumptions, input representation, validation design, and chemical boundaries. Black-box models may still be useful for screening, but their use in lead optimization, formulation strategy, and regulatory-facing decisions requires stronger transparency and validation.

## 8. Challenges and Limitations

Machine learning has expanded the analytical capacity of pharmaceutical chemistry, yet several limitations still restrict its translation into experimental and regulatory practice. The main barriers include data scarcity, variable data quality, weak reproducibility, inconsistent benchmarks, limited experimental validation, and regulatory uncertainty. These issues directly affect model credibility, chemical interpretability, and practical use in molecular design, formulation development, and drug product optimization.

### 8.1. Data Scarcity and Quality

Data scarcity remains a major limitation in pharmaceutical machine learning. Many chemical and formulation datasets are small, proprietary, chemically narrow, or generated under assay-specific conditions. Public datasets are more accessible, but they often contain duplicated compounds, uneven endpoint definitions, missing metadata, and inconsistent experimental protocols [44,92]. These limitations reduce statistical power and increase the risk of overfitting. Data quality is equally important. Solubility, permeability, dissolution, stability, and bioavailability values depend on pH, temperature, salt form, solid state, media composition, assay protocol, and analytical method. If these variables are absent from the dataset, the model may learn incomplete or misleading structure–property associations. In molecular design, this can lead to candidates with favorable predicted properties but poor experimental behavior.

Chemical space coverage also limits generalization. A model trained on narrow scaffold families may perform well under internal validation but fail on new chemotypes. This issue is critical for de novo molecular design, where generated structures may fall outside the training distribution. Data curation, scaffold-aware splitting, uncertainty estimation, and domain-of-applicability analysis are required to reduce these risks [44,77].

### 8.2. Reproducibility Issues

Reproducibility remains a persistent weakness in machine learning studies applied to pharmaceutical chemistry. Many reports lack complete information on dataset preprocessing, descriptor calculation, feature selection, hyperparameter tuning, random seeds, data splitting, and software versions. Without these details, independent verification becomes difficult. Model reproducibility also depends on transparent reporting of negative results and baseline comparisons. Studies often report only the best-performing model, with limited information on failed configurations, alternative representations, or simpler baselines. This may overstate the benefit of complex architectures. A deep learning model should be compared against classical models under identical splits and preprocessing conditions before claims of superiority are made [86,93].

External reproducibility requires accessible datasets and executable code. Proprietary datasets may be unavoidable in industrial pharmaceutical research, but this restricts independent validation. When open data cannot be shared, authors should provide data dictionaries, endpoint definitions, preprocessing protocols, model cards, and validation summaries. These elements improve auditability even when raw data access is limited.

### 8.3. Lack of Standardized Benchmarks

Benchmark inconsistency limits cross-study comparison. Studies often use different datasets, molecular splits, descriptor sets, validation schemes, and performance metrics. Random splitting can inflate performance when structurally related compounds occur in training and test sets. Scaffold splitting provides a stricter assessment of chemical generalization, but it is not applied consistently [44,94]. Metric selection also varies across studies. Regression models may report RMSE, MAE, R^2^, or correlation coefficients, whereas classification models may report accuracy, precision, recall, F1 score, AUROC, or AUPRC. These metrics are not interchangeable. Accuracy can be misleading under class imbalance. R^2^ may obscure pharmaceutically relevant absolute error. Benchmark reporting should match the endpoint and decision task.

Standardized benchmarks are especially needed for integrated SPP mapping. Solubility, permeability, stability, dissolution, and bioavailability are influenced by different experimental conditions. Without harmonized metadata and endpoint definitions, model performance across studies cannot be interpreted reliably. Benchmark design should include chemically meaningful splits, external test sets, uncertainty estimates, and explicit applicability-domain assessment to define where model predictions can be considered reliable [44,94].

### 8.4. Gap Between Prediction and Experimental Validation

A major limitation is the gap between in silico prediction and experimental confirmation. High model performance does not guarantee experimental success. Predicted solubility, permeability, stability, or synthetic feasibility may fail when compounds are tested under real formulation, storage, or biological conditions. This gap is intensified when models are trained on simplified endpoints but applied to complex pharmaceutical performance questions [77,86]. Experimental validation is essential for molecular design. De novo models may generate valid and novel structures, but novelty is not sufficient. Candidates must be assessed for synthetic accessibility, purity, stability, solubility, permeability, toxicity, and biological activity. Without experimental verification, generated molecules remain computational hypotheses [77,86].

The same concern applies to formulation and performance prediction. A dissolution model trained on historical batches must be validated on new formulations, new excipient combinations, and independent manufacturing conditions. A stability model should be tested against stress studies or long-term storage data. A bioavailability model should be evaluated using relevant in vitro, ex vivo, or in vivo evidence. Predictive models should support experimental prioritization rather than replace experimental confirmation.

Several real-world examples illustrate why prospective and experimental validation is essential. In ML-guided molecular design, the identification of potent DDR1 kinase inhibitors demonstrated how computationally prioritized molecules can be synthesized and biologically tested, thereby moving beyond retrospective prediction toward experimental confirmation [8]. In pharmaceutical performance modeling, ML-based prediction of tablet drug-release profiles has shown practical value when formulation variables and release-time data are connected to experimentally measured dissolution behavior [67]. Similarly, constrained neural-network modeling of tablet dissolution slowdown under storage conditions illustrates how ML can be linked with stability-relevant experimental data, humidity effects, and formulation behavior [68]. These examples show that practical ML translation requires more than high internal accuracy. Candidate molecules or formulations must be prospectively synthesized, procured, formulated, characterized, and tested under relevant experimental conditions. The resulting experimental data should then be used to update the model, refine applicability-domain boundaries, and improve confidence in future predictions.

### 8.5. Regulatory Constraints

Regulatory constraints are increasing as AI and ML enter drug discovery, development, manufacturing, and lifecycle management. The European Medicines Agency (EMA) horizon-scanning report identifies AI and ML applications across the medicine lifecycle and highlights opportunities, gaps, and regulatory challenges relevant to future EMA activities [70]. U.S. Food and Drug Administration (FDA) guidance on AI for drug and biological products provides recommendations for AI-generated information used to support regulatory decision-making on safety, effectiveness, or quality, including a risk-based credibility assessment for the model’s context of use [95]. Regulatory acceptability depends on model credibility. A model used for exploratory compound ranking has a lower evidentiary burden than a model used to support a regulatory submission, manufacturing control strategy, or clinical decision. Higher-impact applications require stronger documentation, validation, lifecycle monitoring, and change control. The International Council for Harmonisation (ICH) Q2(R2) also reinforces the broader principle that validated procedures must demonstrate suitability for their intended purpose through defined validation characteristics [96]. Black-box models create specific regulatory difficulty. If a model cannot explain why a compound, formulation, or process condition is predicted to perform well, its use in decision-critical settings becomes limited. Explainability, uncertainty quantification, audit trails, and predefined acceptance criteria are therefore needed. For pharmaceutical chemistry, regulatory readiness requires that ML outputs remain chemically interpretable, experimentally supported, and traceable to defined data sources and validation procedures.

### 8.6. Practical Validation Issues: Dataset Leakage, Molecular-State Effects, Assay Variability, and Uncertainty-Aware Prospective Testing

Beyond general data quality and reproducibility limitations, several practical validation issues are especially important for ML-driven pharmaceutical chemistry and drug discovery. Dataset leakage is a major source of inflated model performance. Random splits can place close analogues, shared scaffolds, replicated molecules, or highly similar assay records across training and test sets, allowing models to exploit chemical similarity rather than learn generalizable structure–property relationships. Scaffold splits are more stringent than random splits, but they are not always sufficient because compounds with different Bemis–Murcko scaffolds may still be close in chemical space. Temporal splits, cluster-based splits, and external test sets provide more realistic estimates of prospective performance, especially when models are intended for lead optimization, virtual screening, ADMET prediction, or formulation decision-making [25,28,44,94].

Assay variability further complicates model comparison and transferability. Solubility, permeability, toxicity, bioavailability, and binding-affinity values may depend on pH, salt form, temperature, cell line, detection method, protein construct, incubation time, and laboratory protocol. If these metadata are missing, models may learn noisy or assay-specific correlations. Therefore, pharmaceutical ML studies should report assay context, remove duplicates carefully, define endpoint units consistently, document preprocessing decisions, and evaluate uncertainty under out-of-distribution conditions. These requirements are especially important for ADMET foundation models and co-folding affinity-prediction models, where benchmark performance may not translate directly to new chemical series, new biological targets, or new assay formats [25,26,27,28,29,30].

Molecular-state effects also require explicit attention. Molecular properties and ligand–protein interactions depend strongly on conformational state, tautomeric form, ionization state, and protonation state. Descriptor-based and SMILES-based models often ignore these effects unless they are explicitly encoded. Even 3D graph, geometric, and equivariant models can be unreliable if conformers are generated under inappropriate protonation or tautomeric assumptions. In structure-based drug discovery, small changes in protonation state can alter hydrogen bonding, electrostatics, binding pose, permeability, solubility, and predicted affinity. These issues become particularly important for joint ligand–protein models and target-conditioned diffusion models, where the generated or predicted complex depends on both ligand geometry and binding-site chemistry [29,30,34]. Future SPP models should therefore report the protonation and tautomer generation protocol, pH assumptions, conformer sampling method, energy filtering criteria, and docking or co-folding pose selection procedure.

Retrospective benchmark performance is insufficient for evaluating pharmaceutical ML models. Prospective validation is needed to determine whether predictions remain reliable for new chemical series, new targets, new formulations, or new assay conditions. Uncertainty-aware workflows can prioritize molecules not only by predicted performance but also by confidence, distance from training data, and expected value of experimental information. In practice, uncertainty should be reported together with prediction intervals, calibration curves, out-of-domain flags, or conformal prediction estimates. Molecules with high predicted performance but high uncertainty should be treated as hypotheses requiring experimental confirmation, not as validated candidates. A robust prospective workflow should include model prediction, uncertainty estimation, synthesis or procurement, orthogonal experimental testing, and feedback into model retraining [32,33,34,77,86,97,98].

In practical pharmaceutical development, prospective validation also faces operational constraints. Predicted compounds may be difficult to synthesize, unstable during purification, poorly soluble in assay media, or incompatible with formulation excipients. Biological validation may be affected by assay format, protein construct, cell line, incubation time, detection method, and batch-to-batch variability. Formulation validation may fail when a model trained on historical batches is applied to new excipient combinations, different processing conditions, or scaled-up manufacturing environments. These translational challenges reinforce the need for stepwise validation, beginning with computational prioritization, followed by synthesis or procurement, analytical confirmation, physicochemical testing, biological or formulation evaluation, uncertainty analysis, and feedback-based model refinement.

Table 10 summarizes the main methodological, validation, and translational challenges affecting ML-driven pharmaceutical chemistry. It integrates the general limitations discussed in Section 8.1, Section 8.2, Section 8.3, Section 8.4 and Section 8.5 with the practical validation issues discussed in Section 8.6, including dataset leakage, assay variability, molecular-state effects, and uncertainty-aware prospective validation.

Table 10 emphasizes that ML models in pharmaceutical chemistry should be evaluated not only by retrospective accuracy, but also by data integrity, reproducibility, chemically meaningful validation design, assay-aware interpretation, uncertainty calibration, prospective testing, and regulatory traceability.

## 9. Future Directions

Future research in machine-learning-driven pharmaceutical chemistry should move from isolated prediction tasks toward experimentally grounded, chemically interpretable, and sustainability-aware design systems. The most important directions include hybrid physics–ML models, generative chemistry, autonomous laboratories, explainable AI, and integration with green chemistry principles.

### 9.1. Hybrid Physics–ML Models

Hybrid physics–ML models represent a strong future direction for pharmaceutical chemistry because they combine empirical data learning with chemically constrained reasoning. Purely data-driven models often perform well within the training distribution, but their reliability decreases when applied to new scaffolds, solvents, solid forms, or formulation conditions. Physics-informed models address this limitation by integrating prior knowledge from thermodynamics, quantum chemistry, molecular mechanics, dissolution theory, transport models, and reaction kinetics [99,100].

In property prediction, hybrid models are especially relevant for solubility, permeability, stability, and drug loading. Recent work on gemcitabine-loaded nanocomposites used curated experimental formulation data and physics-informed synthetic data to predict loading efficiency and encapsulation efficiency [99]. Machine learning combined with physicochemical and computational descriptors has also been used for solubility prediction in organic solvents and water, supporting the broader role of hybrid data-driven and chemistry-informed approaches in pharmaceutical property modeling [100]. The future value of hybrid models lies in better extrapolation. Models constrained by mass balance, solvation principles, diffusion behavior, degradation kinetics, or molecular interaction rules are more likely to provide chemically plausible predictions. This is important for regulatory-facing work, where predictions must remain traceable to scientific assumptions and experimentally verifiable mechanisms.

### 9.2. Generative Chemistry and Inverse Design

Generative chemistry will remain central to ML-driven molecular design. Variational autoencoders, generative adversarial networks, reinforcement learning, graph generative models, diffusion models, and transformer-based molecular language models support de novo design, scaffold modification, and multi-objective molecular optimization [77,78]. Recent advances in generative modeling have significantly expanded the scope of de novo molecular design by enabling the exploration of continuous and high-dimensional chemical spaces. Variational autoencoders and generative adversarial networks map discrete molecular structures into latent representations, allowing for smooth interpolation and property-guided optimization of candidate molecules [79,80]. More recently, diffusion-based models have demonstrated strong capability in generating three-dimensional molecular structures, including conformations compatible with specific protein-binding environments, thereby improving the relevance of generated candidates for structure-based drug design [50].

Recent generative molecular design has moved beyond conventional variational autoencoders (VAEs), generative adversarial networks (GANs), and reinforcement-learning workflows toward latent-space optimization and diffusion-based molecular generation. MolMIM-like latent-space approaches and latent reinforcement-learning workflows support controlled small-molecule generation around desired property constraints, enabling seed-based molecular optimization while preserving chemical similarity and drug-like constraints [31]. This approach is useful for seed-based molecular optimization because structurally related molecules can be explored in latent space while preserving chemical similarity and drug-like constraints.

In parallel, diffusion-based molecular generation has become especially important for 3D and structure-based drug design because it can model atomic coordinates, conformational distributions, and target-conditioned ligand placement. Recent reviews emphasize that diffusion models are increasingly used for 3D molecular generation, target-specific design, conformer generation, molecular docking support, and fragment-based drug design [32,33]. Target-aware E(3)-equivariant diffusion models further strengthen this direction by preserving geometric consistency under rotation and translation while incorporating binding-pocket and property-guidance constraints. DiffGui, for example, integrates atom diffusion, bond diffusion, and property guidance to generate target-conditioned 3D molecules with improved structural feasibility and drug-like properties [34].

Despite these advances, generative performance should not be assessed only by validity, novelty, uniqueness, or retrospective benchmark scores. Generated molecules must also be evaluated for synthetic accessibility, chemical stability, protonation and tautomeric plausibility, conformer quality, predicted ADMET liabilities, binding-site compatibility, and experimental tractability. Target-conditioned diffusion models may propose plausible binding geometries, but unrealistic bond geometry, strained conformations, incorrect protonation states, or poor synthetic feasibility can still limit practical value. Therefore, the next generation of generative SPP workflows should combine MolMIM-like latent optimization, diffusion/equivariant 3D generation, retrosynthetic filtering, uncertainty estimation, and prospective synthesis or biological testing [31,32,33,34].

Across these approaches, constraint-driven design has emerged as a critical requirement, where objective functions incorporate multiple criteria such as pharmacophore preservation, avoidance of toxicophores, scaffold diversity, synthetic accessibility, and target-specific physicochemical properties. These constraints are typically implemented through reward functions, conditional generation, or multi-objective optimization frameworks, ensuring that generated molecules are not only novel but also chemically meaningful, synthetically tractable, and aligned with downstream performance requirements within the structure–property–performance paradigm. Inverse design extends this concept by starting from a desired property or performance profile and searching for structures predicted to satisfy it. The next stage of generative chemistry should focus on constrained design. Generated molecules must satisfy potency, solubility, permeability, metabolic stability, toxicity, synthetic accessibility, and sustainability constraints. Validity and novelty are insufficient. A generated structure has limited value if it is unstable, synthetically impractical, poorly soluble, or inconsistent with the intended route of administration [77,78]. Future generative chemistry should be evaluated through prospective synthesis and experimental testing, not only retrospective benchmark performance. The most useful direction is a closed design loop in which generated molecules are ranked, synthesized, tested, and fed back into model refinement.

Overall, emerging generative approaches should be interpreted as candidate-prioritization and hypothesis-generation tools rather than standalone discovery engines; their pharmaceutical value depends on integration with retrosynthetic analysis, ADMET filtering, uncertainty estimation, and prospective experimental validation.

### 9.3. Autonomous Laboratories

Autonomous laboratories are expected to accelerate chemical discovery by linking prediction, synthesis, characterization, and model updating in closed experimental loops. These systems combine robotics, automated synthesis, analytical instrumentation, experiment planning algorithms, and machine-learning-based decision-making. Their strength lies in reducing the delay between computational prediction and experimental validation. Recent reviews describe autonomous laboratories as systems that combine automation and AI to plan, execute, analyze, and update experiments across chemistry, material science, and biological research [97,98]. These platforms are directly relevant to pharmaceutical chemistry because molecule generation, reaction optimization, formulation screening, and stability testing all require rapid experimental feedback. For pharmaceutical applications, future autonomous laboratories should support synthesis route selection, salt and solid-form screening, formulation optimization, dissolution testing, degradation profiling, and bioassay prioritization. Their impact will depend on data standards, robotic reliability, assay reproducibility, and integration with interpretable ML models.

### 9.4. Explainable AI for Chemical Discovery

Explainable AI will be essential for reliable chemical discovery. Molecular design decisions require more than numerical prediction. They require an explanation of why a structural modification improves solubility, reduces degradation risk, increases permeability, or improves molecular performance. SHAP, feature attribution, attention analysis, atom attribution, counterfactual explanations, and concept-based graph models are expected to become routine in chemical ML workflows. Recent reviews emphasize that explainable AI connects predictive modeling with mechanistic understanding in drug discovery and supports transparency in model-guided decisions [63,87]. In pharmaceutical chemistry, the strongest explanations will be those that align with established chemical reasoning. A solubility model should identify polarity, ionization, hydrogen bonding, and hydrophobic surface area as relevant drivers. A stability model should identify hydrolysis-prone bonds, oxidation-sensitive motifs, photolabile groups, and excipient-related risks. Future work should move beyond post hoc explanation alone. Self-interpretable architectures, chemistry-aware attribution, and mechanism-guided model constraints are needed. These approaches will help distinguish useful chemical explanations from statistical artifacts.

### 9.5. Integration with Green Chemistry

Green chemistry should become a core requirement in ML-driven pharmaceutical design. Molecular optimization should not focus only on potency, solubility, permeability, and stability. It should also consider synthetic route efficiency, solvent safety, reagent hazard, catalyst choice, atom economy, energy demand, waste generation, and environmental persistence. Machine learning is increasingly applied to solvent selection, reaction optimization, catalyst discovery, process design, and sustainable manufacturing. Recent work on drug solubility in green solvents illustrates how ML models can support continuous pharmaceutical manufacturing and environmentally safer solvent systems [101]. Broader AI applications in drug delivery also show increasing movement toward integrated design strategies involving performance, manufacturability, and delivery constraints [87]. Future SPP models should include sustainability descriptors alongside molecular and pharmaceutical endpoints. A candidate molecule should be evaluated not only for biological and developability performance, but also for synthesis burden and environmental cost. This direction links pharmaceutical chemistry with responsible innovation and sustainable drug development.

## 10. Conclusions

Machine learning has transitioned from a purely predictive tool toward a framework capable of capturing chemically meaningful relationships that are consistent with established principles of molecular behavior. Rather than optimizing numerical accuracy alone, effective models increasingly reveal structure-driven determinants that align with physicochemical reasoning and experimental observables. This shift enhances the practical utility of ML in pharmaceutical chemistry, particularly for guiding rational molecular modification and formulation decisions. The structure–property–performance (SPP) paradigm provides a unifying conceptual framework that integrates multiple levels of chemical understanding. It connects molecular design with intermediate physicochemical properties and, ultimately, with measurable performance outcomes such as dissolution, stability, and bioavailability. By linking these domains within a coherent workflow, SPP-based approaches bridge the gap between theoretical prediction and real-world pharmaceutical behavior, enabling more reliable and synthesis-aware decision-making. Future progress in ML-driven pharmaceutical chemistry will depend on the convergence of interpretability and experimental validation. Models must not only produce accurate predictions but also provide chemically consistent explanations that can be verified through laboratory studies. The integration of explainable AI techniques with robust validation strategies will be essential to ensure trust, regulatory acceptance, and practical impact. In this context, the long-term value of machine learning lies in its ability to support transparent, experimentally grounded, and chemically coherent design systems.

## Figures and Tables

**Table 1:** Inclusion and exclusion criteria applied during study selection.

Category	Criteria
Inclusion	Studies addressing chemical or pharmaceutical problems including molecular design, formulation, or process optimization
Inclusion	Application of machine learning or artificial intelligence methods
Inclusion	Explicit description of molecular descriptors, fingerprints, graph, or string representations
Inclusion	Defined validation protocol including data split or cross-validation
Inclusion	Quantitative performance metrics reported
Inclusion	Peer-reviewed full-text articles in English
Exclusion	No chemical or pharmaceutical relevance
Exclusion	No machine learning component
Exclusion	Missing descriptor or preprocessing details
Exclusion	No validation strategy or metrics
Exclusion	Editorials, abstracts, or non-peer-reviewed sources
Exclusion	Insufficient technical detail for evaluation

**Table 2:** Data extraction variables and technical descriptors used for cross-study comparison.

Category	Extracted Variables
Study metadata	Author, year, journal, application domain
Task type	Molecular design, retrosynthesis, formulation, property prediction
Data source	Experimental data, public datasets, simulation, process telemetry
Molecular representation	Descriptors, fingerprints, graphs, SMILES, SELFIES
Tokenization details	Vocabulary, sequence length, padding, truncation
Model type	SVM, Random Forest, Gradient Boosting, CNN, RNN, Transformer, GNN
Architecture details	Layers, hidden dimensions, attention mechanisms
Training strategy	Data split, cross-validation, external validation
Evaluation metrics	Accuracy, precision, recall, F1, AUROC, RMSE, MAE, R^2^
Preprocessing	Normalization, encoding, feature engineering, imbalance handling
Ablation evidence	Full model vs. reduced variants comparison
Output type	Classification or regression endpoints

Note: SVM, support vector machine; CNN, convolutional neural network; RNN, recurrent neural network; GNN, graph neural network; AUROC, area under the receiver operating characteristic curve; RMSE, root mean square error; MAE, mean absolute error; R^2^, coefficient of determination.

**Table 3:** Comparison of molecular representations and their chemical interpretability.

Molecular Representation	Encoded Chemical Information	Typical ML Compatibility	Chemical Interpretability	Main Strengths	Main Limitations	Best Suited Applications
Physicochemical descriptors	Molecular weight, logP, PSA, HBD, HBA, rotatable bonds, charge, polarizability	SVM, Random Forest, XGBoost, MLP, linear models	High	Direct chemical meaning; easy comparison across compounds; suitable for small and medium datasets	Limited structural detail; weak encoding of topology and stereochemistry; depends on predefined descriptor set	Solubility, permeability, lipophilicity, ADME, formulation property prediction
Molecular fingerprints	Presence or absence of substructures, fragments, atom environments	Random Forest, SVM, gradient boosting, neural networks	Moderate	Efficient encoding of substructure patterns; good for similarity search and QSAR modeling	Bit collisions possible; reduced mechanistic clarity; fragment radius affects performance	QSAR, activity prediction, similarity screening, lead optimization
SMILES strings	Atom order, bonds, branches, rings, aromaticity in linear string form	RNN, LSTM, CNN, Transformer, language models	Moderate to low	Compact; widely supported; compatible with large chemical datasets	Multiple strings for one molecule; invalid strings possible; chemical meaning depends on tokenization	Molecular generation, property prediction, reaction prediction, pretrained chemical language models
SELFIES strings	Grammar-constrained molecular tokens with valid decoding rules	RNN, Transformer, generative models, reinforcement-learning models	Moderate	Generated strings decode into valid molecular structures; useful for molecular design	Longer sequences; less intuitive to chemists; less common than SMILES in legacy datasets	De novo design, molecular generation, reinforcement-learning-based optimization
Molecular graphs	Atoms as nodes; bonds as edges; connectivity, topology, atom and bond features	GCN, GAT, MPNN, Graph Transformer	High	Preserves molecular topology; captures local chemical environments; suitable for structure-sensitive prediction	Higher computational cost; requires graph-specific models; long-range interactions need careful model design	Binding prediction, reaction modeling, quantum property prediction, topology-sensitive SPP analysis
3D conformer-based representations	Atomic coordinates, distances, angles, torsions, steric shape, electrostatics	3D GNN, E(n)-equivariant neural networks, geometric deep learning, coordinate-based diffusion models	High	Captures stereochemistry, shape, and spatial interactions	Requires reliable conformer generation; sensitive to protonation and conformational state; higher computational cost	Binding affinity, pharmacophore modeling, chiral property prediction, conformer-dependent activity
Reaction-based representations	Reactants, products, reagents, atom mapping, reaction centers	Transformer, graph models, sequence-to-sequence models	Moderate to high	Captures synthetic feasibility and transformation logic	Requires curated reaction data; atom mapping errors affect quality	Retrosynthesis, reaction prediction, synthesis planning

Note: GCN, graph convolutional network; GAT, graph attention network; MPNN, message passing neural network; MLP, multilayer perceptron.

**Table 4:** Chemical determinants of physicochemical properties and their machine learning interpretation.

Property	Key Chemical Determinants	Common Descriptors	Suitable ML Models	Interpretation Methods
Solubility	Polarity, ionization, hydrogen bonding, crystal packing	PSA, HBD, HBA, logP, MW, charge	RF, XGBoost, ANN, GNN	Feature importance, SHAP
Lipophilicity	Hydrophobic fragments, heteroatoms, ionization, aromaticity	logP, logD, fragment counts, ring descriptors	RF, SVM, XGBoost, GNN	SHAP, fragment attribution
Permeability	Size, polarity, flexibility, ionization, intramolecular H-bonding	MW, PSA, HBD, HBA, rotatable bonds, pKa	XGBoost, ANN, GNN	SHAP, atom attribution, attention

Note: SPR, structure–property relationship; RF, Random Forest; SVM, Support Vector Machine; GNN, graph neural network; SHAP, Shapley additive explanations; PSA, polar surface area; HBD, hydrogen-bond donor; HBA, hydrogen-bond acceptor; logP, partition coefficient for the neutral form; logD, distribution coefficient at a defined pH.

**Table 5:** Property–performance relationships in pharmaceutical chemistry.

Performance Endpoint	Main Property Drivers	Chemical/Formulation Determinants	Suitable ML Inputs	Recommended ML Models	Interpretation Focus
Dissolution behavior	Solubility, particle size, wettability, solid state	Polarity, ionization, crystal form, excipients, porosity	API descriptors, particle size, excipient ratios, process parameters, release time points	RF, XGBoost, ANN, time-series models	Drivers of release rate and profile shape
Stability/degradation	Chemical reactivity, moisture sensitivity, thermal stress	Labile groups, pH, oxygen, humidity, light, packaging	Structural alerts, storage conditions, degradation data, impurity profiles	RF, XGBoost, ANN, constrained neural networks	Degradation pathways and stress sensitivity
Bioavailability	Solubility, dissolution, permeability, metabolism	logD, pKa, PSA, HBD/HBA, transporter liability, formulation type	Molecular descriptors, dissolution data, permeability data, PK variables	Gradient boosting, ANN, hybrid mechanistic–ML models	Exposure-limiting step and absorption risk
Drug performance optimization	Multi-property balance	Potency, solubility, permeability, stability, toxicity	Multi-endpoint datasets, molecular graphs, formulation variables	Multi-task learning, GNN, Bayesian optimization	Trade-off between efficacy and developability

Note: ML, machine learning; API, active pharmaceutical ingredient; RF, Random Forest; ANN, artificial neural network; GNN, graph neural network; PK, pharmacokinetic; logD, distribution coefficient at a defined pH; pKa, acid dissociation constant; PSA, polar surface area; HBD, hydrogen-bond donor; HBA, hydrogen-bond acceptor.

**Table 6:** Multimodal fusion strategies for SPP modeling in pharmaceutical chemistry.

Fusion Strategy	Main Concept	Typical Input Structure	Main Advantage	Key Limitation	Pharmaceutical Use
Early fusion	Concatenates all variables before model training	Molecular descriptors or fingerprints plus tabular formulation/process variables	Simple, transparent, and compatible with RF, XGBoost, SVM, and ANN models	Less suitable for raw graphs, SMILES/SELFIES, images, or 3D conformers without prior encoding	Solubility, dissolution, stability, and formulation screening using curated tabular datasets
Intermediate fusion	Combines learned embeddings from modality-specific encoders	GNN molecular embedding, transformer SMILES/SELFIES embedding, and tabular process embedding	Captures interactions between molecular structure, formulation variables, and process conditions	Requires larger datasets, careful architecture design, and stronger validation	Multimodal SPP prediction, dissolution profiles, degradation risk, ADMET-linked performance
Late fusion	Combines predictions from independently trained models	Separate molecular, formulation/process, and experimental prediction models	Useful when modalities differ in scale, quality, or availability	Interactions between modalities are learned only at the decision level	Ensemble prediction, model stacking, decision-support workflows, uncertainty-aware candidate ranking

**Table 7:** Generative and foundation-model approaches for molecular design.

Approach	Core Capability	Main Use in Molecular Design	Key Limitation	Recommended Validation
Variational autoencoders	Encode and decode molecules through continuous latent space	Latent-space sampling, interpolation, and property-guided molecular generation	May generate low-quality or invalid molecules without chemical constraints	Validity, novelty, uniqueness, synthetic accessibility, ADMET filtering, and prospective testing
Generative adversarial networks	Generate molecules through adversarial training	Distribution matching and de novo candidate generation	Training instability and mode collapse	Chemical-validity checks, diversity analysis, synthetic feasibility assessment, and external property prediction
Diffusion models	Generate structures through iterative denoising	3D molecular generation, conformer generation, target-conditioned design, and structure-based drug discovery	Computationally demanding; validation is complex	Geometry checks, conformer quality assessment, docking or binding validation, ADMET filtering, and synthetic feasibility assessment
Flow-based models	Learn invertible mappings between molecules and latent variables	Controlled sampling and likelihood-based molecular generation	Representation design can be difficult	Likelihood evaluation, validity assessment, scaffold diversity analysis, and external test-set validation
Reinforcement learning	Optimize molecules using reward functions	Multi-objective optimization for potency, selectivity, ADMET properties, and synthesizability	Reward hacking and generation of unrealistic candidates	Reward-function audit, chemical-constraint filtering, retrosynthetic assessment, and prospective experimental confirmation
Genetic algorithms	Mutate, recombine, and select molecules	Search-based molecular optimization and scaffold exploration	Depends strongly on fitness-function design	Fitness-function sensitivity analysis, diversity evaluation, synthetic accessibility checking, and experimental prioritization
ChemBERTa and MoLFORMER	Learn transferable molecular representations from large molecular corpora	Property prediction, virtual screening, similarity analysis, and embeddings for generative workflows	Requires careful fine-tuning, benchmark design, and external validation	Scaffold, time, or cluster split; external ADMET test set; uncertainty calibration
Generative large language models	Link chemical language, text, and molecular strings	Chemical text mining, prompt-guided design, molecule explanation, text-to-molecule translation, and retrosynthetic support	May produce unsupported, chemically unrealistic, or experimentally unvalidated outputs	Chemical validity checking, literature verification, retrosynthetic filtering, ADMET evaluation, uncertainty analysis, and prospective validation

**Table 8:** Frontier machine learning frameworks in pharmaceutical chemistry: capabilities, limitations, and validation needs.

Frontier Approach	Main Capability	Strength	Key Limitation	Recommended Validation
Molecular foundation models, including MolE, ChemFM, SPMM, ChemBERTa/ChemBERTa-3, and MoLFORMER-based models.	Transferable molecular representation and ADMET/property prediction	Useful for low-data and multi-task prediction	Sensitive to benchmark design, endpoint noise, chemical overlap, and domain shift	Scaffold, time, or cluster split; external ADMET test set; uncertainty calibration
MolMIM-like latent-space generation	Seed-based and property-guided molecule generation	Efficient exploration around drug-like molecules	May optimize scoring functions without ensuring synthesis or assay success	Retrosynthesis filtering, ADMET filtering, prospective synthesis
Diffusion-based molecular generation	2D/3D molecular generation and target-conditioned design	Strong for 3D geometry and structure-based molecular design	May generate strained, synthetically difficult, or incorrectly protonated structures	Geometry checks, docking or free-energy validation, synthesis feasibility assessment
Geometric and E(3)-equivariant models	3D molecular and protein–ligand representation	Captures spatial interactions, stereochemistry, and conformer-sensitive effects	Sensitive to conformer quality, protonation state, and pose assumptions	Multiple conformers, pH-aware protonation, external binding datasets
Co-folding and Boltz-2-like models	Joint protein–ligand structure and affinity prediction	Integrates target structure with ligand modeling	Affinity prediction remains sensitive to target state, ligand state, assay context, and training-set overlap	Orthogonal docking, molecular dynamics, free-energy calculations, biochemical assay validation
Uncertainty-aware prospective ML	Confidence-aware candidate prioritization	Reduces overconfident predictions outside training domain	Requires calibration, domain-of-applicability analysis, and experimental feedback	Prospective testing, active learning, calibration analysis

**Table 9:** Interpretability methods for chemically meaningful machine learning.

Interpretability Method	Applicable Model Type	Output Format	Chemical Use	Main Limitation
Feature importance	RF, Gradient Boosting, XGBoost	Ranked descriptors or variables	Identifies important physicochemical drivers such as logP, PSA, HBD/HBA	Correlated descriptors may distort ranking
SHAP	Tree models, ANN, tabular ML	Local and global contribution values	Explains how descriptors increase or decrease predicted solubility, permeability, or stability	Depends on background distribution, descriptor correlation, and input representation; does not prove causality
Attention maps	Transformers, sequence models	Token-level attention weights	Highlights SMILES tokens, functional groups, or reaction-related regions	Attention is not direct mechanistic proof and may reflect statistical association rather than causal chemical importance
Atom or bond attribution	GNN, graph models	Atom-level or bond-level contribution maps	Identifies substructures linked to predicted property or activity	Sensitive to attribution method, graph featurization, perturbation strategy, and model architecture
Concept-based explanations	Self-interpretable GNN, concept models	Human-defined or learned chemical concepts	Links predictions to motifs, fragments, or molecular concepts	Requires careful concept definition and validation

Note: RF, Random Forest; XGBoost, extreme gradient boosting; ANN, artificial neural network; SHAP, Shapley additive explanations; PSA, polar surface area; HBD, hydrogen-bond donor; HBA, hydrogen-bond acceptor; GNN, graph neural network.

**Table 10:** Key methodological, validation, and translational challenges in ML-driven pharmaceutical chemistry.

Challenge	Main Issue	Consequence for ML Studies	Recommended Mitigation
Data scarcity and quality	Small, biased, incomplete, proprietary, or assay-specific datasets	Overfitting, weak generalization, and misleading SPR/SPP conclusions	Data curation, metadata reporting, uncertainty estimation, and domain-of-applicability analysis
Reproducibility	Incomplete reporting of preprocessing, descriptors, data splits, hyperparameters, random seeds, and software versions	Difficult independent verification and inflated model-performance claims	Report code, data splits, model settings, random seeds, software versions, and baseline comparisons
Benchmark inconsistency	Different datasets, endpoints, validation schemes, and performance metrics across studies	Poor cross-study comparability and unreliable claims of model superiority	Use standardized datasets, scaffold/time/cluster splits, external validation, and harmonized metrics
Prediction–experiment gap	In silico outputs are not confirmed experimentally	Computational candidates may fail in synthesis, formulation, biological testing, or stability assessment	Prospective synthesis or procurement, analytical confirmation, orthogonal biological/physicochemical assays, formulation and stability testing, uncertainty analysis, and feedback-based model refinement.
Regulatory constraints	Limited transparency, traceability, intended-use documentation, and lifecycle monitoring	Reduced acceptability in regulatory or decision-critical pharmaceutical settings	Risk-based validation, audit trails, model documentation, explainability, and lifecycle monitoring
Dataset leakage and split strategy	Random splits may place close analogues, duplicated compounds, or highly similar assay records across training and test sets	Inflated performance and weak prospective generalization	Use scaffold, temporal, cluster-based, and external validation splits
Assay variability	Endpoint values depend on pH, salt form, cell line, protein construct, protocol, and laboratory conditions	Noisy or assay-specific correlations that may not transfer to new datasets	Report assay metadata, harmonize endpoints, remove duplicates carefully, and validate across independent assays
Conformational/protonation effects	Molecular state, tautomer form, ionization, and conformer choice may be ignored	Misleading property, pose, affinity, solubility, permeability, or stability predictions	Report pH assumptions, protonation/tautomer protocols, conformer generation, and pose-selection criteria
Uncertainty-aware prospective validation	Retrospective accuracy does not prove reliability for new compounds, targets, formulations, or assay conditions	Overconfident predictions outside the training domain	Use uncertainty calibration, prediction intervals, conformal prediction, domain-of-applicability analysis, and prospective testing

## Data Availability

No new data were generated in this review. All data discussed are available in the cited literature.

## Supplementary Materials

The following supporting information can be downloaded at https://www.mdpi.com/article/10.3390/molecules31122162/s1, Table S1: Full database search strategies used for literature retrieval.

## Abbreviations

The following abbreviations are used in this manuscript:

AI	Artificial Intelligence
ADMET	Absorption, Distribution, Metabolism, Excretion, and Toxicity
ANN	Artificial Neural Network
API	Active Pharmaceutical Ingredient
AUPRC	Area Under the Precision–Recall Curve
AUROC	Area Under the Receiver Operating Characteristic Curve
BERT	Bidirectional Encoder Representations from Transformers
CNN	Convolutional Neural Network
EMA	European Medicines Agency
FDA	Food and Drug Administration
GAN	Generative Adversarial Network
GAT	Graph Attention Network
GCN	Graph Convolutional Network
GNN	Graph Neural Network
HBA	Hydrogen-Bond Acceptor
HBD	Hydrogen-Bond Donor
HOMO	Highest Occupied Molecular Orbital
ICH	International Council for Harmonisation
MAE	Mean Absolute Error
MeSH	Medical Subject Headings
ML	Machine Learning
MLP	Multilayer Perceptron
MPNN	Message Passing Neural Network
MW	Molecular Weight
LUMO	Lowest Unoccupied Molecular Orbital
PK	Pharmacokinetic
PPR	Property–Performance Relationship
PRISMA	Preferred Reporting Items for Systematic Reviews and Meta-Analyses
PRISMA-S	PRISMA Search Reporting Extension
PSA	Polar Surface Area
QSAR	Quantitative Structure–Activity Relationship
QSPR	Quantitative Structure–Property Relationship
RF	Random Forest
RMSE	Root Mean Square Error
RNN	Recurrent Neural Network
SELFIES	Self-Referencing Embedded Strings
SHAP	Shapley Additive Explanations
SMILES	Simplified Molecular Input Line Entry System
SPP	Structure–Property–Performance
SPR	Structure–Property Relationship
SVM	Support Vector Machine
USPTO	United States Patent and Trademark Office
VAE	Variational Autoencoder
XAI	Explainable Artificial Intelligence
XGBoost	Extreme Gradient Boosting
