# Supplementary material for: Machine-Learning-Driven Molecular Design and Structure–Property–Performance Relationships in Pharmaceutical Chemistry
Source: Molecules. 2026 Jun 19;31(12):2162. doi: 10.3390/molecules31122162 (PMC13304929; doi:10.3390/molecules31122162)
Supplement: Supplementary file 1 [file molecules-31-02162-s001.zip › molecules-4355455-supplementary.pdf]

**Table S1.** Full database search strategies used for literature retrieval

| Database | Search String | Boolean Logic Applied | Filters Applied |
|---|---|---|---|
| **PubMed** | ((formulation*[Title/Abstract] OR pharmaceutic*[Title/Abstract] OR "dosage form*"[Title/Abstract] OR excipient*[Title/Abstract] OR dissolution[Title/Abstract] OR stability[Title/Abstract] OR bioavailability[Title/Abstract] OR "molecular design"[Title/Abstract] OR "de novo design"[Title/Abstract] OR "molecular generation"[Title/Abstract] OR "inverse design"[Title/Abstract] OR retrosynthes*[Title/Abstract] OR "chemical synthesis"[Title/Abstract] OR "structure-property relationship*"[Title/Abstract] OR "structure-activity relationship*"[Title/Abstract] OR QSAR[Title/Abstract] OR cheminformatics[Title/Abstract] OR "molecular descriptor*"[Title/Abstract] OR fingerprint*[Title/Abstract] OR SMILES[Title/Abstract] OR SELFIES[Title/Abstract]) AND ("machine learning"[Title/Abstract] OR "artificial intelligence"[Title/Abstract] OR "deep learning"[Title/Abstract] OR "neural network*"[Title/Abstract] OR "support vector machine*"[Title/Abstract] OR "random forest*"[Title/Abstract] OR "gradient boosting"[Title/Abstract] OR XGBoost[Title/Abstract] OR "Bayesian optimization"[Title/Abstract] OR "Gaussian process*"[Title/Abstract] OR "active learning"[Title/Abstract] OR "transfer learning"[Title/Abstract] OR transformer*[Title/Abstract] OR "graph neural network*"[Title/Abstract] OR interpretab*[Title/Abstract] OR "explainable artificial intelligence"[Title/Abstract])) | Two concept blocks combined using **AND**: pharmaceutical/chemical/molecular design terms AND machine learning terms. Synonyms were grouped using **OR**. Field restriction was applied to Title/Abstract. | No filters applied during database search. English-language restriction was applied during screening. |

| | | | |
|---|---|---|---|
| **Web of Science Core Collection** | TS=((formulation* OR pharmaceutic* OR "dosage form*" OR excipient* OR dissolution OR stability OR bioavailability OR "drug delivery" OR "molecular design" OR "de novo design" OR "molecular generation" OR "inverse design" OR "lead optimization" OR retrosynthes* OR "chemical synthesis" OR "structure-property relationship*" OR "structure-activity relationship*" OR SAR OR QSAR OR cheminformatics OR "molecular descriptor*" OR fingerprint* OR SMILES OR SELFIES) AND ("machine learning" OR "artificial intelligence" OR "deep learning" OR "neural network*" OR "support vector machine*" OR "random forest*" OR "gradient boosting" OR XGBoost OR "Bayesian optimization" OR "Gaussian process*" OR "active learning" OR "transfer learning" OR transformer* OR "graph neural network*" OR interpretab* OR "explainable artificial intelligence")) | Topic Search field was used. Two concept blocks were combined using **AND**. Synonyms were grouped using **OR**. Wildcards were applied for term expansion. | No filters applied during database search. English-language restriction was applied during screening. |
| **Scopus** | TITLE-ABS-KEY((formulation* OR pharmaceutic* OR "dosage form*" OR excipient* OR dissolution OR stability OR bioavailability OR "drug delivery" OR "molecular design" OR "de novo design" OR "molecular generation" OR "inverse design" OR "lead optimization" OR retrosynthes* OR "chemical synthesis" OR "structure-property relationship*" OR "structure-activity relationship*" OR SAR OR QSAR OR cheminformatics OR "molecular descriptor*" OR fingerprint* OR SMILES OR SELFIES) AND ("machine learning" OR "artificial intelligence" OR "deep learning" OR "neural network*" OR "support vector machine*" OR "random forest*" OR "gradient boosting" OR XGBoost OR "Bayesian optimization" OR "Gaussian process*" OR "active learning" OR "transfer learning" OR transformer* OR "graph neural network*" OR interpretab* OR "explainable artificial intelligence")) | TITLE-ABS-KEY fields were used to retrieve records from titles, abstracts, and keywords. Two concept blocks were combined using **AND**. Synonyms were grouped using **OR**. Scopus was included to broaden coverage of chemical, pharmaceutical, and interdisciplinary literature. | No filters applied during database search. English-language restriction was applied during screening. |

| | | | |
|---|---|---|---|
| **IEEE Xplore** | (("molecular design" OR "drug design" OR "de novo design" OR "molecular generation" OR "inverse design" OR "retrosynthesis" OR "reaction prediction" OR "chemical synthesis" OR "QSAR" OR "structure-activity relationship" OR "structure-property relationship" OR "cheminformatics" OR "molecular representation" OR "molecular descriptor" OR "fingerprint" OR "SMILES" OR "SELFIES") AND ("machine learning" OR "artificial intelligence" OR "deep learning" OR "neural network" OR "graph neural network" OR "transformer" OR "generative model" OR "variational autoencoder" OR "VAE" OR "generative adversarial network" OR "GAN" OR "reinforcement learning" OR "diffusion model" OR "Bayesian optimization" OR "Gaussian process" OR "active learning" OR "transfer learning")) | Two concept blocks were combined using **AND**: molecular design/cheminformatics terms AND machine learning/generative modeling terms. The search emphasized algorithmic and computational studies. | No filters applied during database search. English-language restriction was applied during screening. |
| **ChemRxiv** | ("machine learning" OR "artificial intelligence" OR "deep learning" OR "graph neural network" OR transformer OR "generative model" OR "Bayesian optimization" OR "active learning") AND ("molecular design" OR "de novo design" OR "molecular generation" OR "retrosynthesis" OR "reaction prediction" OR "chemical synthesis" OR "structure-property relationship" OR QSAR OR ADMET OR solubility OR permeability OR bioavailability) | Simplified keyword-based Boolean searches were used because ChemRxiv search functions are less structured than bibliographic databases. ChemRxiv was used as a supplementary source to identify recent preprints in chemical sciences and emerging ML-based molecular design methods. | No filters applied during retrieval. During screening, ChemRxiv records were included only when they showed clear pharmaceutical chemistry relevance, sufficient methodological detail, and an identifiable validation strategy. |

**Note:** The search strategies were designed according to PRISMA-S recommendations, using database-specific syntax, explicit Boolean logic, and unrestricted retrieval to maximize sensitivity. Eligibility restrictions, including English language, peer-review status, methodological completeness, pharmaceutical relevance, and validation quality, were applied during screening rather than during database search.
